# Toward a critical dialectical pluralist methodology: operationalizing the integrated mixed methods transformation approach via a representational–recursive framework

**DOI:** 10.3389/fpsyg.2026.1907909

**Published:** 2026-10-21

**Authors:** Anthony J. Onwuegbuzie, Sandra Schamroth Abrams

**Affiliations:** 1 Department of Educational Leadership and Management, University of Johannesburg, Johannesburg, South Africa; 2 School of Education, The University of Sheffield, Sheffield, United Kingdom; 3 Department of Psychology of Education, University of South Africa, Pretoria, South Africa

**Keywords:** critical dialectical pluralism (CDP) 2.0, integrated mixed methods transformation approach (IMMTA), integration, meta-inference, methodological theory, mixed methods research, recursion, representational analysis

## Abstract

Mixed methods research continues to privilege additive and sequential forms of integration, limiting its capacity to generate genuinely transformative and emergent knowledge. The Integrated Mixed Methods Transformation Approach (IMMTA), grounded in Critical Dialectical Pluralism (CDP) 2.0, offers a meta-transformative alternative but remains analytically under-specified. We address this limitation by advancing a representational–recursive framework that reconceptualizes data as analytic representations and integration as recursive re-representation (i.e., transformatizing). We cluster 26 IMMTAs into five domains—epistemic plurality, transformative integration, reflexivity and positionality, justice-oriented inquiry, and emergence and complexity—and demonstrate how these domains are operationalized through representational ontology, recursive analytic cycles, and meta-inferential processes. This synthesis yields a unified methodological system in which integration emerges through iterative interaction rather than aggregation. Throughout this article, the term *emergence* is used in two related but distinct senses. First, emergence refers to the complexity-theoretic property whereby system-level understandings arise from interactions among multiple analytic components and cannot be reduced to any single component alone. Second, emergence refers to the practical methodological process through which increasingly refined interpretations arise during recursive cycles of representation, integration, and meta-inference. Although related, the former denotes an epistemological property of integrated inquiry, whereas the latter denotes the analytic process through which such understandings develop. The framework advances mixed methods methodology by specifying mechanisms of transformation, enhancing analytic rigor, and repositioning integration as an emergent, system-level property of inquiry, with particular relevance for investigating complex psychological phenomena.

## From partial integration to transformative mixed methods inquiry

Mixed methods research has undergone substantial evolution over the past several decades, moving from early efforts focused on combining qualitative research and quantitative research methods toward more sophisticated conceptualizations of integration. Despite these advances, a central challenge persists: integration still is enacted frequently as an additive or procedural feature of research design rather than as a fundamentally transformative process. In this article, we advocate for the *Integrated Mixed Methods Transformation Approach* (IMMTA; Onwuegbuzie and Abrams, 2026), which intentionally focuses on the process of integration, an approach that is essential for transforming, advancing, and innovating research and research designs.

### Why IMMTA?

Although mixed methods research in the psychological sciences tends to be disseminated across a range of publications and, thus, might not seem to be a popular methodology, it is one that is used by psychological researchers despite misconceptions or reservations (Creamer and Reeping, 2020; Syed and Wilkinson Westberg, 2025). In many mixed methods research studies, qualitative research and quantitative research components/phases/strands are conducted in parallel or in sequence. In these instances, integration typically occurs at limited junctures—often through *triangulation* (i.e., comparing findings from the qualitative data with the quantitative results), *complementarity* (i.e., seeking elaboration, illustration, enhancement, and clarification of the findings from one analytical component/phase/strand [e.g., qualitative] with results from the other analytical component/phase/strand [e.g., quantitative]), *development* (i.e., using the results from one analytical component/phase/strand to help inform the other analytical component/phase/strand), *initiation* (i.e., discovering paradoxes and contradictions that emerge when findings from the two analytical phases/strands are compared that might lead to a re-framing of the research question), and e*xpansion* (i.e., expanding the breadth and range of a study by using multiple analytical component/phase/strand for different study phases) (Greene et al., 1989). Although such approaches can yield valuable insights, they rarely achieve the level of conceptual, analytic, and epistemological synthesis required to address complex, multidimensional phenomena, including *wicked problems* (i.e., problems involving multiple interacting systems, social and institutional uncertainties, and no clear solutions; Mertens et al., 2016) and *super wicked problems* (i.e., problems that intensify these features through heightened urgency, partner entanglement, and systemic constraints; Levin et al., 2012).

This limitation reflects, in part, the enduring influence of methodological dualisms that position qualitative research and quantitative research approaches as distinct, and at times competing, paradigms/traditions. These dichotomies—along with related distinctions such as subjective versus objective, emic versus etic, micro versus macro, perspective versus truth, universals versus particulars, facts versus values, relativism versus absolutism, and constructivist versus realist—continue to shape how integration is conceptualized and enacted. As a result, even within mixed methods research, integration often is constrained by underlying assumptions that reify difference rather than enabling synthesis (Onwuegbuzie and Hitchcock, 2019). Addressing this challenge requires not only methodological innovation, but also a rethinking of the philosophical foundations that undergird mixed methods inquiry.

Mixed methods methodology has been shaped by several influential philosophical and methodological traditions that provide different rationales for integration. Creswell and Plano Clark (2007, 2011, 2018) conceptualize mixed methods research primarily through research designs and procedural integration strategies, emphasizing the intentional coordination of qualitative research and quantitative research approaches across stages of inquiry. Instead, Morgan (2007, 2014) advances a pragmatist perspective in which methodological choices are guided by the practical consequences of inquiry and the research questions being addressed rather than by adherence to any single philosophical paradigm. Mertens (2003, 2007, 2010, 2012) argues from a transformative-emancipatory paradigm that foregrounds social justice, equity, human rights, and the inclusion of marginalized communities as central purposes of mixed methods inquiry. More recently, critical mixed methods scholars have emphasized reflexivity, power, representation, complexity, and the ethical implications of integration, arguing that methodological innovation should extend beyond procedural design toward critical engagement with knowledge production itself (e.g., Younas et al., 2026). Collectively, these perspectives substantially have advanced mixed methods methodology by demonstrating that integration encompasses not only methodological procedures, but also philosophical commitments, ethical orientations, and epistemological assumptions.

These developments also have given rise to several ongoing debates within contemporary mixed methods methodology that extend beyond the traditional qualitative–quantitative divide. Increasing attention has been directed toward questions concerning the nature and depth of integration, the relationship between philosophical paradigms and methodological practice, the role of reflexivity and researcher positionality, the ethical implications of knowledge production, the treatment of complexity and emergence, and the extent to which mixed methods inquiry should be viewed as a design strategy, a methodological orientation, or a broader philosophical enterprise (Creamer, 2018; Fetters and Freshwater, 2015; Greene, 2007; Johnson, 2023; Morgan, 2007, 2014; Poth, 2018, 2022). Rather than positioning IMMTA as a response only to historical qualitative–quantitative dichotomies, the present framework is intended to contribute to these broader methodological conversations by proposing representational recursion and transformatizing as mechanisms through which integration can become more fully recursive, theoretically grounded, ethically responsive, and methodologically transformative.

Critical Dialectical Pluralism (CDP) 2.0 (Onwuegbuzie and Abrams, 2025) offers such a foundation. Because the philosophical foundations of CDP 2.0 and IMMTA have been presented in detail elsewhere, our discussion here intentionally is concise and limited to concepts necessary for understanding the operational framework developed in the remainder of this article. As a meta-transformative and multidimensional metaparadigm and meta-philosophical framework, CDP 2.0 is grounded in three interrelated meta-principles: criticality, dialectics, and pluralism. Criticality foregrounds issues of power, marginalization, and representation, emphasizing the need for researchers to engage with questions of equity and justice. Dialectics emphasizes the productive potential of tensions, contradictions, and differences, positioning synthesis as an emergent outcome of interaction rather than as a predefined endpoint. Pluralism affirms the legitimacy of multiple ways of knowing, rejecting hierarchical or exclusionary epistemologies in favor of inclusive and dialogical approaches to inquiry. Together, these principles provide a coherent philosophical basis for reimagining mixed methods research as a dynamic, reflexive, and socially responsive enterprise.

Building on this foundation, IMMTA advances a meta-transformative vision of mixed methods research. Rather than conceptualizing integration as the combination of discrete methodological components, IMMTA frames integration as a process of transformation that occurs across all stages of the research process. In this view, qualitative research and quantitative research approaches are not treated as separate strands to be linked or merged, but as interrelated and co-evolving elements of a unified inquiry. IMMTA thus represents a significant shift from additive and partially integrative models toward a conception of mixed methods research as an inherently *integrative* (i.e., characterized by the dynamic interaction of qualitative research and quantitative research approaches throughout the research process), *integrated* (i.e., involving the systematic and coherent interweaving of methodological components across all stages of inquiry), and *integral* (i.e., constituting a unified and inseparable whole in which methodological distinctions are subsumed within a holistic analytic system) form of inquiry, as well as a holistic and emergent approach. In practice, mixed methods research in general, and IMMTA, in particular, can add analytic dimension to psychological studies. As Syed and Wilkinson Westberg (2025) contend, content validity and triangulation improve when qualitative and quantitative data inform each other. Although these concepts are closely related, they refer to distinct levels of methodological organization: *integrative* denotes the ongoing process of interaction, *integrated* denotes the coordinated implementation of that interaction across the research process, and *integral* denotes the resulting holistic unity of the methodological system.

However, despite its conceptual promise, IMMTA currently lacks a fully articulated analytic infrastructure that can guide its implementation in empirical research. Although the framework identifies multiple pathways for transforming monomethod approaches into integrated mixed methods research designs, it does not yet specify the mechanisms through which integration unfolds at the level of data, analysis, and inference. Consequently, researchers seeking to apply IMMTA might encounter challenges in translating its meta-transformative principles into concrete methodological practices.

In this article, we address this gap by developing a representational–recursive framework that operationalizes IMMTA within the philosophical grounding of CDP 2.0. Importantly, although this article builds directly on our earlier exposition of CDP 2.0 and IMMTA, its purpose fundamentally is different. The earlier work introduced the philosophical and methodological foundations of CDP 2.0 and IMMTA. In contrast, the present article advances the research program by operationalizing IMMTA through a representational–recursive analytic framework, organizing the 26 IMMTAs into five higher order conceptual domains, specifying mechanisms through which integration unfolds recursively across the research process, and illustrating how these mechanisms can be enacted across diverse methodological contexts in psychology. Thus, rather than reiterating the earlier framework, this article extends it by providing the analytic infrastructure necessary for its practical implementation.

The present framework is intended to complement rather than replace these established perspectives. Whereas Creswell and Plano Clark (2007, 2011, 2018) primarily emphasize design typologies and procedural integration, Morgan (2007, 2014) highlights pragmatic decision making, and Mertens (2003, 2007, 2010, 2012) foregrounds transformative social justice commitments, IMMTA focuses specifically on the analytic mechanisms through which integration unfolds recursively during knowledge construction. Similarly, recent critical mixed methods frameworks have emphasized reflexivity, power, and representation; IMMTA builds on these developments by proposing representational recursion and transformatizing as explicit mechanisms for operationalizing transformative integration. Accordingly, IMMTA should be viewed as participating in an ongoing dialogue within mixed methods methodology rather than as superseding alternative approaches.

Because several concepts developed in this article emerge from an ongoing methodological research program, we have sought to make the present account fully self-contained. Accordingly, all concepts essential to the argument—including IMMTA, transformatizing, representational ontology, recursive analytic cycles, and meta-inference—are defined, explained, illustrated, and theoretically justified within this article rather than requiring readers to consult unpublished or forthcoming works. Thus, readers can evaluate the conceptual coherence, methodological rationale, and practical implications of the proposed framework independently of our related scholarship. Citations to our prior or related scholarship are used to establish intellectual provenance and continuity, whereas the substantive warrant for the present framework is developed through direct argumentation and engagement with the wider mixed methods research literature.

More specifically, although the present article builds on concepts introduced in our broader methodological research program, it does not assume the validity of those concepts merely because they have been proposed elsewhere. Rather, IMMTA, transformatizing, and the representational–recursive framework are developed here through explicit conceptual definitions, theoretical argumentation, engagement with the broader mixed methods methodology literature, and illustrations of their methodological utility. Consequently, the present article is intended to be evaluated on the internal coherence, explanatory power, and practical usefulness of its arguments rather than on the authority of our prior publications. In this sense, references to related works document the intellectual evolution of the research program rather than serving as circular justification for the framework advanced herein. Accordingly, readers need not accept the proposed framework on the basis of its prior publication history; rather, its merits should be judged by the conceptual coherence, explanatory power, methodological transparency, and practical utility developed within the present article.

In so doing, IMMTAs offer practical guidance for researchers wishing to achieve integration throughout the research process. The IMMTA framework is based on the premise that what are commonly referred to as *data* are more appropriately understood as *analytic representations*—constructed through processes of selection, interpretation, and transformation (Cabrera and Reiner, 2018; Collingridge, 2013; Driscoll et al., 2007; Isaac et al., 2016; Kerrigan, 2014; Leal et al., 2018; Miles and Huberman, 1994; Sandelowski et al., 2009; Tashakkori and Teddlie, 1998; Weaver-Hightower, 2014). From this perspective, integration is not achieved by combining fixed entities but by engaging in recursive cycles of re-representation, wherein qualitative and quantitative forms continuously are transformed and reinterpreted in relation to one another. This process is referred to as transformatizing (Onwuegbuzie, 2026), emphasizing its dynamic, multidirectional, and iterative character.

In order to avoid conceptual ambiguity, we distinguish transformatizing from related concepts in the mixed methods research literature. Whereas quantitizing (Cabrera and Reiner, 2018; Collingridge, 2013; Driscoll et al., 2007; Isaac et al., 2016; Kerrigan, 2014; Leal et al., 2018; Miles and Huberman, 1994; Sandelowski et al., 2009; Tashakkori and Teddlie, 1998; Weaver-Hightower, 2014) and qualitizing (Tashakkori and Teddlie, 1998) involve transforming one form of data into another (e.g., qualitative data into quantitative form or vice versa), transformatizing refers to the broader recursive process through which analytic representations are repeatedly re-represented, reinterpreted, integrated, and refined throughout the research process. Likewise, although crossover analyses involve the intentional integration of qualitative and quantitative analyses, typically, they denote particular analytic procedures or points of integration. In contrast, transformatizing characterizes an overarching meta-process in which successive cycles of representation, interpretation, integration, and meta-inference continually reshape both the evolving analysis and the emerging understanding of the phenomenon under investigation.

Importantly, transformatizing, therefore, should not be understood simply as another form of data conversion or crossover analysis. Rather, quantitizing, qualitizing, and crossover analyses can each function as specific analytic operations within a broader transformatizing process. Thus, whereas these established approaches describe particular transformations or points of integration, transformatizing denotes the recursive methodological architecture that continually reorganizes representations, interpretations, and meta-inferences throughout the entire research process.

In order to facilitate the operationalization of IMMTA, we organize the 26 Integrated Mixed Methods Transformation Approaches into five higher order conceptual clusters: *epistemic plurality*, *transformative integration*, *reflexivity and positionality*, *justice-oriented inquiry*, and *emergence and complexity*. These clusters provide a parsimonious structure for understanding the diverse ways in which transformation can be enacted within mixed methods research. Then, we demonstrate how the representational–recursive framework aligns with and extends these clusters by providing analytic mechanisms for transformation, iteration, and emergent meta-inference.

Table 1 presents the 26 IMMTAs organized within these five conceptual clusters, alongside their primary alignment with the core principles of CDP 2.0. In addition, the table distinguishes the underlying monomethod approaches, methods, or techniques from their corresponding IMMTAs, identifies key developers or foundational contributors, and provides summary descriptions that illustrate how each transformation is operationalized in practice. The table is not intended as a rigid or mutually exclusive classification; rather, it provides a heuristic mapping that highlights the dominant orientation of each approach while acknowledging their overlap and interdependence. Importantly, the 26 IMMTAs should not be interpreted as claims to entirely new or to replacement methodologies. Rather, they represent meta-transformative reconceptualizations of established approaches, methods, and techniques through the philosophical lens of CDP 2.0 and the operational framework of representational–recursive integration. Thus, the innovation resides not in renaming familiar methodologies such as ethnography, phenomenology, systematic reviews, or narrative inquiry, but in specifying how these approaches can be transformed into fully integrated mixed methods research in which recursive integration, transformatizing, and meta-inference occur throughout the entire research process rather than at isolated stages. Accordingly, the IMMTAs are intended to provide an *operational taxonomy of transformative possibilities* rather than a taxonomy of wholly new methodologies. In order to enhance scholarly transparency, it is important to distinguish the locus of innovation represented by the IMMTAs. With very few exceptions, the IMMTAs are not proposed as entirely new methodologies independent of prior methodological traditions. Rather, each IMMTA represents a meta-transformative extension of an established research approach, method, or technique that has been reconceptualized through the philosophical commitments of CDP 2.0 and operationalized through recursive representational integration. Consequently, the novelty resides primarily in the integrated methodological architecture—the recursive, representational, and meta-inferential processes through which qualitative and quantitative inquiry become fully integrated throughout the research process—rather than in replacing or renaming existing methodologies. In this sense, the table functions as a bridge between philosophical commitments and methodological enactment, illustrating how criticality, dialectics, and pluralism are instantiated across diverse forms of inquiry. By making these alignments explicit, Table 1 offers a parsimonious yet integrative framework for understanding how transformation can be operationalized within mixed methods research.

**Table 1:** The 26 integrated mixed methods transformation approaches (IMMTAs) organized by conceptual cluster and linked to CDP 2.0 principles.

Mono method approach, method, or technique	IMMTA	Conceptual cluster	Developers/Foundational contributors	Summary description	Primary CDP principle
Interviews	Mixed Methods Interviews (MMIs)	Epistemic Plurality	Frels and Onwuegbuzie (2013)	Fully integrates structured qualitative interviews with quantitative survey data for deeper participant insights.	Pluralism
Focus group discussions	Mixed Methods Focus Group Discussions (MMFGDs)	Epistemic Plurality	Onwuegbuzie et al. (2009, 2010) and Onwuegbuzie et al. (In Press)	Fully integrates focus group discussion (FGD) data with quantitative measures to enhance FGDs through statistical and thematic analysis for the purpose of examining shared and divergent perspectives.	Dialectics
Ethnography	Mixed Methods Ethnography Research (MMER)	Epistemic Plurality	Crede and Borrego (2013), Hitchcock and Onwuegbuzie (2020), Schensul and LeCompte (2012), and Schensul et al. (2012)	Fully integrates ethnographic observation with quantitative data to contextualize cultural patterns.	Pluralism
Phenomenological Research	Mixed Methods Phenomenology Research (MMPR)	Epistemic Plurality	Mayoh and Onwuegbuzie (2014, 2015)	Fully integrates lived experiences (qualitative) with empirical data (quantitative) to understand phenomena to examine essence and variation of experiences.	Pluralism
Narrative Inquiry	Mixed Methods Narrative Inquiry (MMNI)	Epistemic Plurality	Onwuegbuzie et al. (2024a)	Fully integrates storytelling techniques with quantitative measures to explore meaning-making processes.	Pluralism
Design-based Research	Integrated Mixed Methods Design-Based Research (IMM-DBR)	Transformative Integration	Onwuegbuzie et al. (2023a, 2023b)	Fully integrates iterative design processes with qualitative and quantitative research methodologies to develop, to test, and to refine interventions in real-world settings, ensuring that findings are both theoretically sound and practically applicable.	Dialectics
Research through Design	Integrated Mixed Methods Research Through Design (IMMRD)	Transformative Integration	Onwuegbuzie and Abrams (2026b)	Fully integrates iterative design processes with mixed methods research approaches to develop and to evaluate new innovations.	Dialectics
Research into Design	Integrated Mixed Methods Research into Design (IMMRID)	Transformative Integration	Onwuegbuzie and Abrams (2026b)	Fully integrates mixed methods analysis into the research process of developing new designs and policies	Criticality
Research for Design	Integrated Mixed Methods Research For Design (IMMRFD)	Transformative Integration	Onwuegbuzie and Abrams (2026b)	Applies fully integrated mixed methods research approaches to refine and to optimize the implementation of designed systems and solutions.	Criticality
Autoethnography	Integrated Mixed Methods Autoethnography (IMMA)	Reflexivity & Positionality	Onwuegbuzie et al. (2024a, 2024b)	Fully integrates personal reflexive storytelling with quantitative data to examine personal and societal change.	Criticality
Case Study	Mixed Methods Case Study (MMCS)	Reflexivity & Positionality	Onwuegbuzie and Wisdom (2013), Sharp et al. (2012), and Walton et al. (2020)	Fully integrates multiple data sources within bounded systems for in-depth contextual analysis of complex cases.	Dialectics
Narrative Research	Mixed Methods Narrative Research (MMNR)	Reflexivity & Positionality	Onwuegbuzie and Abrams (2024)	Fully integrates structured narrative analysis with mixed methods research for policy and human behavior studies.	Pluralism
Photovoice	Integrated Mixed Methods Photovoice (IMMPV)	Reflexivity & Positionality	Nguyen and Ngo (2024)	Fully integrates Participatory Action Research (PAR) and Photovoice (PV) to explore their synergies, tensions, and implications.	Criticality
Child–Parent Research	Integrated Mixed Methods Child–Parent Research (IMMCPR)	Justice-Oriented Inquiry	Abrams et al. (2020), Abrams and Schaefer (2022), Schaefer et al. (2020, 2021)	Fully integrates child and parent as co-researchers in dialogic, participatory, and ethically grounded inquiry to co-construct knowledge about meaning making, relationships, and lived experience while working to flatten traditional research hierarchies.	Criticality
Participatory Video Research	Integrated Mixed Methods Participatory Video Research (IMMPVR)	Justice-Oriented Inquiry	Nawrath et al. (2024)	Fully integrates visual/qualitative participatory video data with quantitative environmental and behavioral indicators (e.g., biodiversity measures, ecological metrics, and community participation indices).	Criticality
Participatory Research	Mixed Methods Community-Based Participatory Research (MMCBPR)	Justice-Oriented Inquiry	DeJonckheere et al. (2019)	Fully integrates collaborative qualitative inquiry with quantitative measures for co-produced knowledge to promote social change.	Criticality
Peace Research	Mixed Peace Research	Justice-Oriented Inquiry	Onwuegbuzie and Tashakkori (2015)	Fully integrates qualitative research and quantitative research approaches to study conflict and peacebuilding and to examine peace and conflict, addressing complex socio-political issues through a critical, reflexive, and transformative lens that fosters interdisciplinary engagement and policy relevance.	Criticality
Action Research	Critical Mixed Methods Action Research (CMMAR)	Justice-Oriented Inquiry	Christ (2009), Ivankova (2015), Ivankova and Wingo (2018), and Onwuegbuzie and Dickinson (2007)	Uses fully integrated approaches to apply participatory cycles explicitly toward social justice and transformation.	Criticality
Impact Evaluation	Mixed Methods Impact Evaluation (MMIE)	Justice-Oriented Inquiry	Onwuegbuzie and Benge (2018), Onwuegbuzie and Hitchcock (2017), and White (2009)	Fully integrates quantitative impact assessments with qualitative narratives to assess interventions, treatments, or policies.	Criticality
Visual Displays	Mixed Methods Visual Displays (MMVDs)	Emergence & Complexity	Kleih et al. (2022) and Onwuegbuzie and Dickinson (2008)	Fully integrates visual representation of data with quantitative data and qualitative narratives to improve data communication.	Pluralism
Grounded Theory	Mixed Methods Grounded Theory (MM-GT)	Emergence & Complexity	Creamer (2018, 2021), Guetterman et al. (2017), Howell Smith et al. (2020), Johnson et al. (2010), Johnson and Walsh (2019), and Shim et al. (2021)	Generates and tests theory through iterative integration of qualitative coding and quantitative patterns.	Dialectics
Systematic Reviews	Integrated Mixed Methods Systematic Reviews (IMMSR)	Emergence & Complexity	Onwuegbuzie and Abrams (2026c)	Fully integrates systematic reviews of quantitative and qualitative studies by synthesizing qualitative and quantitative evidence across studies for comprehensive evidence synthesis.	Pluralism
Bibliometrics	Mixed Methods Bibliometrics (MMB)	Emergence & Complexity	Onwuegbuzie and Frels (2016) and Onwuegbuzie et al. (2018)	Fully integrates bibliometric analysis with qualitative interpretation of knowledge trends in order to examine trends in the academic literature.	Pluralism
Randomized Controlled Trials	Mixed Methods Randomized Controlled Trials (RCTs)	Emergence & Complexity	Edmunds et al. (2022)	Fully integrates randomized control trials with qualitative process data to explain causal mechanisms and to evaluate intervention impacts.	Criticality
Quasi-experimental Methods	Quasi-Experimental Mixed Methods (QEMM)	Emergence & Complexity	Nielsen et al. (2017)	Fully integrates experimental and quasi-experimental designs with contextual qualitative insights to evaluate interventions.	Criticality
Assessment	Transformational Assessment	Emergence & Complexity	Onwuegbuzie et al. (2024a)	Fully integrates qualitative research and quantitative research methodologies to examine critically, to reconstruct, and to refine assessment systems, ensuring that they equitably and accurately capture diverse human experiences while challenging systemic biases and power imbalances in research and evaluation.	Criticality

Cluster assignments and CDP 2.0 principle alignments reflect dominant orientations; several IMMTAs span multiple domains, consistent with the pluralistic and dialectical commitments of CDP 2.0.

By integrating CDP 2.0, IMMTA, and representational–recursive analysis, this article advances a unified methodological paradigm for mixed methods research. Accordingly, the present article should be viewed not as a restatement of CDP 2.0 or IMMTA, but as the next stage in their methodological development, translating previously articulated philosophical principles into an operational framework for conducting fully integrated mixed methods research. Consistent with this objective, the present framework is intended to complement, to extend, and to enter into dialogue with existing mixed methods methodologies rather than to replace them. We view methodological advancement as cumulative and dialogical, benefiting from continued comparison, refinement, critique, and application across diverse scholarly perspectives. Although the framework is applicable across disciplines, it has particular relevance for psychological science, wherein researchers increasingly confront complex, multilevel phenomena that require integrative approaches capable of addressing contextual, developmental, social, cognitive, and behavioral dimensions simultaneously. In so doing, it contributes to ongoing efforts to move beyond partial integration—what Fetters and Freshwater (2015) refer to as the “1 + 1 = 3” integration approach, wherein the integration of qualitative research and quantitative research components/phases/strands yields insights that exceed those generated by either approach alone—toward a more comprehensive, reflexive, and justice-oriented form of inquiry, one in which knowledge is not merely combined, but co-constructed through dynamic processes of interaction and transformation.

### Critical dialectical pluralism (CDP) 2.0 as a meta-philosophical foundation

#### Origins and rationale

Critical Dialectical Pluralism (CDP) 2.0 (Onwuegbuzie and Abrams, 2025) emerges from longstanding critiques of research practices that privilege extraction over reciprocity, fragmentation over synthesis, and hierarchy over inclusion. Across disciplines, scholars have highlighted how conventional research traditions often reproduce asymmetrical power relations, wherein participants’ knowledge is appropriated, decontextualized, and reinterpreted through externally imposed frameworks. Such practices not only constrain the scope of inquiry, but also limit the ethical and epistemic integrity of research by marginalizing alternative ways of knowing and privileging dominant perspectives—a phenomenon that Onwuegbuzie and Abrams (2025) characterize as a “lack of attention to within-study social justice” (p. 2).

In response to these concerns, CDP 2.0 advances a meta-transformative and multidimensional metaparadigm and meta-philosophical orientation that reconfigures both the purposes and processes of inquiry. Rather than treating research as a neutral or purely technical endeavor, CDP 2.0 positions it as an inherently value-laden and relational activity, embedded within broader social, cultural, and political contexts. This shift entails a dual transformation. First, it requires an *ethical reorientation*, wherein research explicitly is aligned with principles of social justice, inclusion, diversity, equity, and social responsibility— collectively representing what Onwuegbuzie and Abrams (2025) refer to as the five SIDES pillars of CDP 2.0. Second, it demands an *epistemic transformation*, wherein multiple forms of knowledge are recognized as legitimate and are engaged in ways that resist reduction, hierarchy, or assimilation.

Importantly, CDP 2.0 proponents do not advocate the replacement of one dominant paradigm/tradition with another. Instead, it offers a framework for engaging difference productively through synthesis, dialogue, and reflexive critique (Onwuegbuzie and Abrams, 2025). In this sense, CDP 2.0 provides the philosophical conditions necessary for moving beyond methodological fragmentation toward integrative, integrated, and integral forms of inquiry. It is precisely this capacity—to hold together ethical commitment and epistemic plurality within a coherent framework—that renders CDP 2.0 particularly suited to grounding advances in mixed methods research.

#### The three meta-principles: criticality, dialectics, and pluralism

Although introduced earlier, these meta-principles warrant further elaboration because they provide the conceptual foundation through which CDP 2.0 is operationalized within mixed methods inquiry. At the core of CDP 2.0 are three interrelated meta-principles—criticality, dialectics, and pluralism—which function not as discrete components but as mutually constitutive dimensions of inquiry.

*Criticality* foregrounds the role of power in shaping knowledge production. It calls for sustained attention to issues of marginalization, representation, and voice, requiring researchers to interrogate whose perspectives are privileged, whose are excluded, and how these dynamics influence both the research process and its outcomes. Within CDP 2.0, criticality extends beyond critique as a purely analytical exercise; it is also oriented toward transformation (Cabrera and Reiner, 2018; Collingridge, 2013; Driscoll et al., 2007; Isaac et al., 2016; Kerrigan, 2014; Leal et al., 2018; Miles and Huberman, 1994; Sandelowski et al., 2009; Tashakkori and Teddlie, 1998; Weaver-Hightower, 2014). That is, identifying inequities is inseparable from working to address them, whether through inclusive research design, participatory practices, or the redistribution of interpretive authority.

*Dialectics* provides the mechanism through which difference is engaged rather than resolved prematurely. Rather than viewing tensions—such as those between qualitative research and quantitative research approaches—as obstacles to be overcome, dialectical thinking treats them as generative. Contradictions, divergences, and inconsistencies are not signs of methodological failure but opportunities for deeper understanding. Through iterative interaction, opposing or contrasting perspectives can be brought into relation, yielding insights that neither perspective could produce independently. In this way, synthesis is not imposed but emerges through the dynamic interplay of difference.

*Pluralism* affirms the legitimacy of multiple ways of knowing and resists epistemological hierarchy, thereby contributing to the flattening of hierarchies in knowledge production (see, for, e.g., Abrams et al., 2020). Within CDP 2.0, pluralism does not imply relativism or the absence of evaluation; rather, it entails recognizing that different forms of knowledge may illuminate different dimensions of a phenomenon. Pluralism thus supports the inclusion of diverse epistemologies, methodologies, and analytic strategies, while maintaining a commitment to coherence through integration. It provides the philosophical basis for engaging multiple perspectives without collapsing them into a single dominant framework.

Taken together, these three principles establish a foundation for inquiry that is simultaneously critical, integrative, and inclusive. Criticality ensures that research remains attentive to power and justice; dialectics enables the productive engagement of difference; and pluralism legitimizes the coexistence of multiple forms of knowledge. This triadic structure underpins CDP 2.0 and informs its application across methodological contexts, including mixed methods research.

#### The SIDES framework: ethical operationalization

Although introduced earlier, the SIDES framework warrants further elaboration because it serves as the primary mechanism through which the ethical commitments of CDP 2.0 are translated into research practice. Whereas the three meta-principles articulate the philosophical orientation of CDP 2.0, the SIDES framework—comprising social justice, inclusion, diversity, equity, and social responsibility—provides its ethical operationalization. These dimensions translate abstract commitments into guiding principles for research practice.

*Social justice* directs attention to the broader consequences of research, emphasizing the need to address inequities and contribute to the well-being of individuals and communities. It positions research as a vehicle for positive change rather than as an end in itself (Onwuegbuzie and Abrams, 2025).

*Inclusion* requires that all relevant voices are recognized and valued within the research process. This extends beyond participant recruitment to encompass involvement in decision-making, interpretation, and dissemination, thereby challenging traditional distinctions between researchers and participants (Abrams et al., 2020; Abrams and Schaefer, 2022; Schaefer et al., 2020, 2021).

*Diversity* acknowledges the multiplicity of perspectives, experiences, and contexts that shape knowledge. Within CDP 2.0, diversity is not treated as a variable to be controlled but as a resource that enriches understanding (Onwuegbuzie and Abrams, 2025).

*Equity* moves beyond formal equality to address disparities in power, access, and representation. It involves creating conditions under which all contributors can participate meaningfully and have their knowledge recognized on its own terms (Onwuegbuzie and Abrams, 2025).

*Social responsibility* emphasizes the obligation of researchers to ensure that their work is responsive to societal needs and accountable to the communities that it affects. This includes careful consideration of how findings are used, interpreted, and disseminated.

Together, these five dimensions embed ethical considerations within every stage of the research process. Rather than functioning as external criteria applied after the fact, they are integral to how research is conceptualized, conducted, and evaluated. In this way, the SIDES framework operationalizes the ethical commitments of CDP 2.0, ensuring that methodological choices are aligned with broader goals of justice and inclusion (Onwuegbuzie and Abrams, 2025).

#### CDP 2.0 and the rejection of dichotomies

A defining feature of CDP 2.0 is its explicit rejection of entrenched dichotomies that historically have structured methodological discourse. Among the most influential of these is the distinction between qualitative research and quantitative research. Traditionally, these approaches have been framed as representing fundamentally different epistemological orientations, often leading to their separation in both training and practice. Even within mixed methods research, this divide frequently persists, with integration typically occurring only after qualitative and quantitative components/phases/strands have been developed independently.

CDP 2.0 challenges this binary by reframing qualitative research and quantitative research approaches as complementary rather than as oppositional. From a dialectical perspective, their differences are not barriers to integration but sources of analytic potential. Similarly, the distinction between *subjective* and *objective* is reconsidered. Rather than treating subjectivity as a source of bias and objectivity as an ideal of neutrality, CDP 2.0 recognizes that all knowledge is situated and that reflexive engagement with subjectivity can enhance, rather than undermine, analytic rigor. In this sense, knowledge is understood as intersubjectively constructed, emerging through the dynamic interaction of multiple perspectives rather than residing solely within individual subjectivities or objective structures (Berger and Luckmann, 1967).

The *emic–etic* distinction likewise is reinterpreted. Instead of privileging either insider (i.e., emic) or outsider (i.e., etic) perspectives, CDP 2.0 researchers encourage their interaction, allowing locally grounded understandings and externally informed analyses to inform one another. This reconceptualization aligns with Dwyer and Buckle’s (2009) notion of the *space between*, which challenges binary positioning by enabling researchers to occupy simultaneous insider–outsider roles. Extending this perspective, Onwuegbuzie (2012) introduced the concept of *emtic* to denote the dynamic integration of emic and etic viewpoints, emphasizing their maximal interaction within a unified analytic space. From this standpoint, insider and outsider perspectives are not endpoints on a dichotomy but co-constitutive sources of insight whose interaction yields a more comprehensive and contextually responsive understanding. In each case, the goal is not to eliminate difference, but to engage it productively through dialectical processes that foster integrative, reflexive, and expansive forms of inquiry.

This perspective also is broadly compatible with methodological approaches that emphasize multiplicity and the inevitable partiality of representation, such as Law’s (2004, 2007) conception of *messy* realities. Like Law (2004, 2007), CDP 2.0 rejects the assumption that research can produce a single, complete, or final representation of complex phenomena. Both perspectives acknowledge that inquiry necessarily foregrounds some aspects of phenomena while backgrounding others, and both regard this partiality as an inherent feature of knowledge production rather than as a methodological failure. However, CDP 2.0 differs in placing greater emphasis on the productive role of recursive integration among multiple representations. Whereas Law primarily encourages researchers to remain attentive to multiplicity, ambiguity, and ontological messiness, CDP 2.0 further proposes that dialectical engagement among diverse representations can generate increasingly comprehensive, ethically responsive, and reflexively constructed meta-inferences without implying that these ever become exhaustive or final. Consequently, representational ontology within CDP 2.0 should be understood as complementing rather than replacing perspectives that emphasize ontological multiplicity and methodological uncertainty.

Although CDP 2.0 emphasizes representational ontology, this emphasis should not be interpreted as implying that inquiry is concerned only with representations or that reality itself is reducible to discourse or cognition. Rather, CDP 2.0 operates under the assumption that researchers engage with material, social, cultural, psychological, and institutional realities only through representations that always are partial, situated, and historically contingent. Consequently, epistemological transformation refers to the recursive transformation of how phenomena are understood, represented, interpreted, and acted on rather than to the claim that research directly transforms reality itself. At the same time, because knowledge informs practice, policy, intervention, and social action, transformed understandings can contribute indirectly to transformations of material conditions. Thus, CDP 2.0 researchers distinguish between transforming representations of reality through inquiry and contributing to the transformation of reality through the subsequent application of knowledge. This position avoids both idealism, wherein reality is reduced to thought or discourse, and naïve materialism, wherein representations are assumed to mirror reality directly. Instead, it conceptualizes inquiry as a reflexive, ethically situated process through which increasingly comprehensive representations support more informed engagement with an irreducibly complex and evolving world.

By rejecting rigid dichotomies, CDP 2.0 creates the conditions for a more integrative and flexible approach to inquiry. This shift particularly is significant for mixed methods research, wherein such dichotomies historically have limited the depth of integration (Onwuegbuzie and Abrams, 2025). In moving beyond binary thinking and adopting a synechist (i.e., anti-dualistic) stance (Johnson and Gray, 2010), aligned with Johnson’s (2023) *both/and* logic of combination rather than an *either/or* stance, CDP 2.0 reconceptualizes research as a process in which diverse forms of data, analysis, and interpretation are brought into dynamic relation.

#### Positioning CDP 2.0 as the foundation for IMMTA

Within this meta-transformative and multidimensional framework, CDP 2.0 serves as the meta-philosophical grounding that enables the IMMTA. The meta-principles of criticality, dialectics, and pluralism provide the conceptual basis for understanding integration as a transformative, rather than additive, process. The SIDES framework ensures that such transformation is ethically oriented toward social justice, diversity, inclusion, equity, and social responsibility. The rejection of dichotomies removes structural barriers that historically have constrained integration, opening space for more fluid and dynamic forms of inquiry.

IMMTA builds directly on these foundations by translating them into a methodological framework for mixed methods research. Whereas CDP 2.0 articulates *why* integration should be critical, dialectical, and pluralistic, IMMTA addresses *how* such integration can be enacted across the research process. Thus, CDP 2.0 and IMMTA are not independent frameworks but are best understood as operating at complementary levels: CDP 2.0 as a meta-philosophical orientation and IMMTA as its methodological expression. These principles not only are meta-philosophical commitments, but also are instantiated in methodological approaches. As shown in Table 1, specific IMMTAs operationalize criticality, dialectics, and pluralism in distinct yet overlapping ways.

Although every IMMTA is grounded philosophically in the three meta-principles of CDP 2.0 and the ethical commitments embodied in the SIDES framework, the IMMTAs do not operationalize these principles identically or to the same degree. Rather, each IMMTA represents a different methodological expression of the same overarching meta-philosophical orientation. Differences among the IMMTAs arise not because they rest on competing or incommensurable philosophical assumptions, but because they address different research purposes, contexts, phenomena, analytic challenges, and ethical priorities. For example, justice-oriented IMMTAs naturally foreground criticality and the SIDES commitments of social justice, equity, and inclusion, whereas IMMTAs emphasizing emergence and complexity necessarily place greater emphasis on dialectical interaction, recursive transformation, and pluralistic integration across evolving representations. Likewise, approaches centered on reflexivity and positionality emphasize the continual examination of researcher assumptions while simultaneously engaging criticality, dialectics, and pluralism. Thus, the relative emphasis placed on particular CDP 2.0 principles reflects differences in methodological function rather than differences in philosophical foundation. Therefore, collectively, the IMMTAs should be viewed as being complementary operationalizations of a shared CDP 2.0 worldview rather than as distinct methodological paradigms grounded in incompatible assumptions.

Importantly, CDP 2.0, the SIDES framework, IMMTA, and the representational–recursive framework do not require integration because qualitative research and quantitative research are assumed to be ontologically identical or fully commensurable. Rather, integration is pursued because different forms of representation illuminate different dimensions of complex phenomena while simultaneously revealing the limitations, assumptions, blind spots, and interpretive possibilities of one another. From this perspective, partial incommensurability is not an obstacle to integration but one of its primary justifications. Recursive engagement among diverse representations enables tensions, contradictions, complementarities, and absences to become analytically productive, thereby generating richer and more ethically responsive meta-inferences than would be possible from any single representation alone. Accordingly, integration within IMMTA should not be understood as collapsing qualitative research and quantitative research approaches into a single homogeneous account or eliminating their distinctive ontological and methodological commitments. Instead, it represents a dialectical process through which differences remain visible, are critically interrogated, and recursively inform one another while preserving their respective contributions. Thus, the aim of integration is neither assimilation nor epistemic closure but the continual co-construction of increasingly comprehensive, reflexive, and contextually responsive understandings of complex psychological and social phenomena.

### IMMTA as a meta-transformative methodological framework

#### From mixing to transformation

The evolution of mixed methods research can be understood as a progression in how integration itself is conceptualized. Early formulations emphasized the combination of qualitative research and quantitative research approaches, often represented implicitly as a simple additive logic—1 + 1 = 2. However, in practice, this model frequently involved the parallel implementation of qualitative research and quantitative research components/phases/strands, with findings and interpretations generated and disseminated separately rather than integrated (Bryman, 2006). Although this approach expanded methodological flexibility, it largely preserved the independence of qualitative research and quantitative research components/phases/strands, with little or no integration occurring across stages of the research process.

Subsequent developments moved toward more interactive conceptions of integration, often framed by Fetters and Freshwater (2015) as *1 + 1 = 3*, wherein the combination of methods yields insights that exceed the sum of their parts. This perspective acknowledges the potential for synergistic knowledge production, particularly when qualitative and quantitative findings intentionally are related through triangulation, complementarity, development, initiation or expansion (Greene et al., 1989). However, even in these more integrative approaches, qualitative and quantitative elements frequently remain distinguishable, and their interaction often is structured rather than fully dynamic (Bryman, 2006; Collins, 2018; Creamer, 2018; Fetters et al., 2013; Fetters and Freshwater, 2015; Poth, 2018, 2022).

IMMTA represents a further conceptual shift, captured metaphorically as *1 + 1 = 1* (Onwuegbuzie and Hitchcock, 2019). In this formulation, integration is not the outcome of combining separate components but the defining condition of the research process itself. Qualitative research and quantitative research approaches are no longer treated as independent strands to be linked or merged; instead, they are understood as interdependent, co-constructed, and co-evolving aspects of a unified inquiry. Integration thus becomes continuous, recursive, and transformative, occurring across *all* stages of research process (Onwuegbuzie and Hitchcock, 2019) rather than at predefined points (Fetters et al., 2013). This shift reflects a broader reorientation from *methodological pluralism as coexistence* toward *pluralism as interaction*, wherein the boundaries between approaches are permeable and analytically productive.

Practically, integration is not a linear plan for research. Rather, when developing the research from its inception through its dissemination, the researcher needs to consider how to conceptualize holistically the qualitative data and quantitative data. Questions, such as, “How will the data inform themselves?” challenge the researcher to consider and to plan for data and analysis to take shape in anticipated and unanticipated ways. For example, Abrams’s (2024) integrated mixed methods autoethnography enabled her to pair her quantitative understandings of pain and disability with her qualitative journal entries. As she began to analyze her data, she started to collect additional data and explore visuals to reconceptualize her noticings. She used a word cloud generator, which paired image with statistical frequency outputs. Abrams created a chart that she then annotated, transforming data from their raw state to visual and numerical representations that, in a recursive fashion, became a platform for Abrams not only to engage reflexively with the data, but also to continue collecting, analyzing, and synthesizing the data. Abrams relied on the image of the cycle of water in an aerating pond fountain to articulate how the data transformed, remixed, and evolved in continuous and unexpected ways (see Onwuegbuzie et al., 2024a). In other words, because Abrams did not work with binaries nor did she envision research as linear, she was able to embrace the fluid, recursive nature of the integrated mixed methods transformational approach.

Importantly, recursive integration extends beyond data generation and analysis to encompass the writing, peer discussion, interpretation, dissemination, and subsequent application of findings. Within IMMTA, writing is itself an analytic and integrative activity in which evolving qualitative representations, quantitative representations, visual representations, theoretical insights, and reflexive considerations continue to reshape one another. Likewise, peer review, collaborative interpretation, and dialogue with research participants, community partners, practitioners, policymakers, and other knowledge users constitute additional opportunities for recursive transformation rather than merely external evaluation. Consistent with the critical and dialectical commitments of CDP 2.0, disagreement is not regarded as a methodological problem to be eliminated but as a productive source of further inquiry that can expose hidden assumptions, challenge dominant interpretations, reveal marginalized perspectives, and stimulate the development of more comprehensive and ethically responsive meta-inferences. Moreover, the SIDES framework requires continual attention to power asymmetries throughout these recursive processes by encouraging researchers to examine whose voices are privileged, whose interpretations are marginalized, who participates in interpretive negotiations, and how interpretive authority can be shared more equitably. Thus, integration within IMMTA is understood not as the achievement of consensus or the resolution of all differences but as an ongoing process of critical, dialogical, and ethically accountable knowledge co-construction that remains open to continued reinterpretation as new participants, perspectives, contexts, and representations emerge.

Importantly, this example also illustrates how recursive transformatizing generates meta-inference. Rather than treating qualitative journal entries, quantitative indicators, and visual representations as separate products of analysis, Abrams recursively interpreted each evolving representation in relation to the others until they collectively yielded a more comprehensive understanding of pain, disability, and the research process itself. Thus, transformatizing occurred through iterative cycles of representation, reinterpretation, further data generation, and re-representation, with meta-inference emerging progressively from these recursive interactions rather than from a single final act of integrating qualitative and quantitative findings.

Importantly, this transformation is not merely technical but epistemological. The metaphor of “1 + 1 = 1” should not be interpreted as denying that integration necessarily involves processes of combination, synthesis, or interaction. Rather, it emphasizes that these processes ultimately culminate in the emergence of a unified methodological system whose properties cannot be reduced to its constituent qualitative research and quantitative research components/phases/strands. In this sense, IMMTA does not reject synthesis; instead, it reconceptualizes synthesis as an ongoing, recursive, and transformative process rather than as a discrete event in which two previously independent strands simply are merged. Qualitative and quantitative representations continue to interact throughout the inquiry, but through recursive transformatizing, they progressively lose their status as analytically independent entities and, instead, function as mutually constitutive dimensions of a single evolving system of inquiry. Accordingly, the expression “1 + 1 = 1” is intended as an ontological and epistemological metaphor that foregrounds the emergence of methodological unity rather than the arithmetic combination of separate methods. It entails a rethinking of what constitutes *data*, how meaning is generated, and how knowledge claims are constructed. By foregrounding *transformation* rather than *combination*, IMMTA aligns with perspectives that emphasize emergence, reflexivity, and the co-construction of knowledge, thereby extending the trajectory of mixed methods research beyond additive and even synergistic models of integration.

The overall goal of IMMTA is not to eliminate plurality, difference, or ongoing recursion by producing a single final or exhaustive representation of reality. Rather, its purpose is to generate increasingly comprehensive, contextually responsive, ethically accountable, and practically useful understandings of complex phenomena through the recursive interaction of multiple representations. Therefore, integration is not pursued because researchers assume that disparate perspectives ultimately converge on a complete or definitive account. Instead, integration is valuable because recursive engagement among multiple representations can reveal relationships, tensions, contradictions, and emergent insights that remain inaccessible when representations are considered in isolation. The resulting meta-inferences always are provisional, revisable, and situated, remaining open to further transformation as new representations, contexts, and perspectives emerge. Thus, IMMTA conceptualizes integration as an ongoing process of knowledge development rather than as the achievement of epistemic closure.

#### Defining IMMTA

As noted previously, the IMMTA can be defined as a meta-transformative methodological framework that reconceptualizes mixed methods research as an inherently integrative and dynamic process. At its core, IMMTA shifts the focus from the coordination of distinct methodological components to the transformation of inquiry itself. This transformation operates along two interrelated dimensions: the movement from monomethod to mixed methods research and the extension of integration across all stages of the research process.

First, IMMTA reframes the transition from monomethod research to mixed methods research as a process of transformation rather than supplementation. In traditional designs, mixed methods research approaches often are constructed by adding a secondary method to primary one—for example, incorporating qualitative research approaches into a predominantly quantitative study, or incorporating quantitative research approaches into a predominantly qualitative study. IMMTA challenges this additive logic by emphasizing that the introduction of multiple methods fundamentally should reshape the research design, including its questions, data generation strategies, analytic procedures, and interpretive frameworks. In this sense, mixed methods research is not simply “more” than monomethod research (i.e., *1 + 1 = 3*); it is qualitatively different, requiring a reconfiguration of the relationships among all elements of inquiry.

Second, IMMTA posits that integration should occur *across all stages of the research process*, including conceptualization, design, data collection, analysis, interpretation, and dissemination. Rather than being confined to a specific phase—such as the integration of results—IMMTA treats integration as an ongoing and recursive process. Decisions made at one stage inform and are informed by decisions at other stages, creating a dynamic interplay that allows for continuous refinement and transformation. This perspective aligns with calls for more fully integrated mixed methods research designs, in which qualitative and quantitative components are not only combined, but also interwoven throughout the research process (Creamer, 2018).

A key implication of this approach is that integration is no longer viewed as a discrete methodological task, but as a defining feature of the research process itself. IMMTA thus repositions integration from the periphery to the center of mixed methods inquiry, making it the primary mechanism through which knowledge is generated. In so doing, it provides a methodological expression of the principles articulated in CDP 2.0, particularly the emphasis on dialectical interaction and epistemic plurality (Onwuegbuzie and Abrams, 2025).

#### Clustering the 26 IMMTAs

In order to facilitate the application of IMMTA in research practice, the 26 Integrated Mixed Methods Transformation Approaches (IMMTAs) can be organized into the following five higher order conceptual clusters: epistemic plurality, transformative integration, reflexivity and positionality, justice-oriented inquiry, and emergence and complexity. This clustering provides a parsimonious structure for understanding the diverse pathways through which transformation can occur, while preserving the richness and specificity of individual approaches. Although categorized, each IMMTA can exist across clusters, and each IMMTA reflects one or more CDP 2.0 principles, linking philosophical orientation to methodological practice (see Table 1).

#### Epistemic plurality

The first cluster emphasizes the recognition and inclusion of multiple ways of knowing. IMMTAs within this domain foreground the legitimacy of diverse epistemologies, methodological traditions, and analytic perspectives. Rather than privileging a single framework, researchers using these IMMTAs fully integrate different forms of knowledge in ways that respect their distinct contributions while enabling their interaction. This cluster reflects the pluralist dimension of CDP 2.0 and supports the development of research designs that are responsive to complex, multifaceted phenomena. The following five IMMTAs fall under this cluster: Mixed Methods Interviews (MMIs), Mixed Methods Focus Group Discussions (MMFGDs), Mixed Methods Ethnography Research (MMER), Mixed Methods Phenomenology Research (MMPR), and Mixed Methods Narrative Inquiry (MMNI) (see Table 1).

For example, Mixed Methods Focus Group Discussions (MMFGDs) fully integrate focus group discussion data with quantitative measures through both thematic and statistical analyses, thereby enabling the examination of shared and divergent perspectives within and across groups. In so doing, MMFGDs preserve the richness of interactive, co-constructed qualitative meaning while simultaneously identifying patterns and variations across participants, allowing individual voices and collective dynamics to inform one another without reduction (Onwuegbuzie et al., In Press).

Although some examples of research with focus group discussions that include mixed methods highlight the aspect of transformation, there remains a parsing of qualitative data and quantitative data into strands, likely due to the adoption of the 1 + 1 = 3 approach to integration (e.g., Vogl, 2019). As such, and especially in this instance, transformation is not part of each step in the research process (i.e., from design through dissemination; cf. Cabrera and Reiner, 2018; Collingridge, 2013; Driscoll et al., 2007; Isaac et al., 2016; Kerrigan, 2014; Leal et al., 2018; Miles and Huberman, 1994; Sandelowski et al., 2009; Tashakkori and Teddlie, 1998; Weaver-Hightower, 2014); rather, transformation is a feature that is rendered as a separate entity that stems from the merging of the qualitative strand and the quantitative strand during data analysis. Achieving a full(er) transformation and integration (Creamer, 2018), as is the case with MMFGDs, involves a reconceptualization of the research from its inception. Vogl perceived the evolving nature of data collection and how it can inform different analytical approaches, which is an important component to achieve an IMMTA. Yet, researchers interested in MMFGDs that involve full(er) integration and transformation would need a flexible methodological mindset from the inception of the research to support epistemic plurality and various ways of knowing that can emerge through an integrated transformational approach wherein the data are holistically conceptualized and connected, instead of parsed and patched together at particular moments in the research design.

Similarly, extant research that involves mixed methods narrative inquiry appears to represent mixed methods in/and narrative inquiry. For example, Waller et al. (2021) engaged with qualitative data and quantitative data sequentially, relying on the former to inform the latter to develop a narrative about father involvement in child wellbeing. Although the authors conducted a mixed methods narrative analysis, integration occurred only at the analysis stage and with the qualitative data and the quantitative data addressed in different stages. Whereas this is one type of integration, IMMTA means that integration and transformation occur throughout the study, not piecemeal or at particular stages (i.e., the analysis stage).

Mixed Methods Narrative Inquiry (MMNI) fully integrates storytelling techniques with quantitative measures to explore meaning-making processes. This approach honors the depth, temporality, and contextual specificity of narrative accounts (Waller et al., 2021) while using quantitative data to illuminate broader patterns, relationships, or distributions, thereby enabling multiple layers of understanding to coexist and to interact (Onwuegbuzie and Abrams, 2024). Such an approach involves intentionality when researchers design their study, wherein they address how qualitative data and quantitative data will work in concert in such a way that neither type of data is perceived nor treated as a separate entity. Rather, from the onset of the design, inclusive of a conceptual orientation for a full(er) integration (i.e., 1 + 1 = 1), researchers would develop research questions and data collection techniques that holistically engage qualitative data and quantitative data. In this way, transformation also can occur conceptually as well as practically. In both cases, integration is achieved not through homogenization but through the relational coordination of distinct knowledge forms, making these approaches particularly well suited to addressing complex, multifaceted phenomena.

#### Transformative integration

The second cluster focuses on the processes through which integration becomes transformative. IMMTAs in this category emphasize the movement beyond additive or sequential designs toward full(er) integrated approaches in which qualitative elements and quantitative elements are interwoven. Transformation occurs through iterative interaction, reinterpretation, and the reconfiguration of analytic categories. This cluster captures the core methodological innovation of IMMTA, positioning integration as a dynamic and generative process. The following four IMMTAs fall under this cluster: Integrated Mixed Methods Design-Based Research (IMM-DBR), Integrated Mixed Methods Research Through Design (IMMRD), Integrated Mixed Methods Research into Design (IMMRID), and Integrated Mixed Methods Research for Design (IMMRFD) (see Table 1).

Extant research is moving towards full(er) integration. For example, Claravall et al. (2026) engaged in Transformative MM-DBR to explore equitable literacy instruction for special education students. The authors embraced “a recursive process where multiple data sources continuously inform the development of literacy intervention” and wherein the practitioner and the university researcher’s collective feedback shaped the intervention (p. 6). Focusing on the equity inherent in transformative research, Claravall et al. (2026) underscored how the Transformative MM-DBR equally supported the voices of the practitioners and their university partners. However, the approaches differ from IMM-DBR.

Although Claravall et al. included the collection of qualitative data and quantitative data simultaneously, the data were analyzed separately first and then engaged in a mixed analysis. In an Integrated Mixed Methods Design-Based Research (IMM-DBR) study, quantitative data and qualitative data are not treated separately at any point in the research. For example, IMM-DBR fully integrates iterative design processes with qualitative research and quantitative research methodologies to develop, to test, and to refine interventions in real-world settings. Within this approach, transformation occurs through iterative cycles in which qualitative insights (e.g., participant experiences, contextual observations) and quantitative findings (e.g., performance metrics, outcome measures) continuously are brought into interaction to inform successive design refinements. These cycles facilitate reinterpretation by allowing emerging qualitative themes to reshape the meaning of quantitative results and, conversely, by using quantitative patterns to recontextualize qualitative interpretations. In turn, analytic categories are reconfigured as initial constructs are revised, expanded, or integrated in response to insights generated across iterations. Thus, integration is not confined to a single phase of analysis but unfolds recursively throughout the design process, yielding increasingly refined, contextually responsive, and theoretically informed understandings (Onwuegbuzie et al., 2023a, 2023b).

#### Reflexivity and positionality

The third cluster highlights the role of the researcher and the importance of reflexive engagement with positionality (Alvesson and Sköldberg, 2009; Berger, 2015; Finlay, 2002; Hesse-Biber and Leavy, 2007). Although each of the IMMTAs could be subsumed into this cluster, we highlight four IMMTAs within this domain that help to emphasize that researchers are not neutral observers but active participants in the construction of knowledge. Reflexivity involves ongoing examination of how researchers’ perspectives, assumptions, and interactions shape the research process. By incorporating reflexivity into integration, this cluster ensures that transformation is not only methodological (Cabrera and Reiner, 2018; Collingridge, 2013; Driscoll et al., 2007; Isaac et al., 2016; Kerrigan, 2014; Leal et al., 2018; Miles and Huberman, 1994; Sandelowski et al., 2009; Tashakkori and Teddlie, 1998; Weaver-Hightower, 2014), but also epistemological. The following four IMMTAs fall under this cluster: Integrated Mixed Methods Autoethnography (IMMA), Mixed Methods Case Study (MMCS), Mixed Methods Narrative Research (MMNR), and Integrated Mixed Methods Photovoice (IMMPV) (see Table 1).

For example, Integrated Mixed Methods Autoethnography (IMMA) fully integrates personal reflexive storytelling (Ellis et al., 2011) with quantitative data to examine personal and societal change (Onwuegbuzie et al., 2024a, 2024b). Within this approach, reflexivity is not treated as a supplementary analytic step but as a central mechanism of integration, wherein the researcher’s lived experiences, positionality, and interpretive lenses are brought into continuous dialogue with quantitative findings. This interaction enables the reinterpretation of both narrative and numerical data, as personal insights shape the meaning of statistical patterns while quantitative results, in turn, prompt deeper reflection on lived experience. In this way, analytic categories are not fixed but are co-constructed and recursively refined through the interplay of self-reflection and empirical analysis.

In their mixed method autoethnography, Clubbs and Chin (2024) pair the dynamic p-technique of structural equation modeling with autoethnographic reflexive journaling in an effort to “‘put the person’ back into [the] center of scholarship and inquiry” (p. 464). The design of their study intentionally integrated quantitative data collection and analysis with qualitative data collection and analysis, inclusive of the second author engaging in the quantitative and qualitative analysis and auditing of the data the first author collected. Taking one step further, with IMMA, transformation extends beyond methodological integration (Cabrera and Reiner, 2018; Collingridge, 2013; Driscoll et al., 2007; Isaac et al., 2016; Kerrigan, 2014; Leal et al., 2018; Miles and Huberman, 1994; Sandelowski et al., 2009; Tashakkori and Teddlie, 1998; Weaver-Hightower, 2014) to encompass epistemological reconfiguration, as knowledge is generated through the reflexive engagement of the researcher as both subject and analyst.

Similarly, mixed methods case study has begun to show signs of full(er) integration. Researchers (e.g., Plano Clark et al., 2018) have highlighted the affordances of mixed methods case study research, identifying the ways that the synergies of qualitative research and quantitative research can help to address complex, real-world issues. Yet, Plano Clark et al. also identify how mixed methods case study research can fall short due to partial integration because researchers treated quantitative research and qualitative research as tangential (i.e., collected together but analyzed separately) and as embedded (i.e., nesting one within the other, thereby privileging one method over another). The authors begin to unpack the importance of integration in their discussion of mixed methods case study that highlights the “intersection” of “assumptions, intents, logics, and methods of mixed methods research and case study research in order to more completely describe and interpret the complexity and theoretical importance of a case or cases” (p. 20). Given that Plano Clark et al. advocate for integration at every level, their work helps to exemplify how MMCS might take shape, inclusive of researcher acknowledgement of philosophical positions. We extend this discussion by further emphasizing the need for greater researcher reflexivity and positionality beyond philosophical stance to account for existing and evolving researcher biases at every stage of the research process (Berger, 2015; Finlay, 2002). Such reflexivity and positionality are hallmarks of IMMCS.

#### Justice-oriented inquiry

The fourth cluster aligns with the ethical commitments of CDP 2.0, particularly those articulated in the SIDES framework. IMMTAs in this category emphasize the role of research in addressing issues of power, inequality, and social justice. Although research in other clusters might have a social justice mission or lens (e.g., IMM-DBR), we parse justice-oriented inquiry to underscore how integration is oriented toward amplifying underserved, marginalized, oppressed, disenfranchised, and silenced voices. As such, through a full(er) integration approach, researchers can challenge dominant narratives, and producing knowledge that contributes to equitable outcomes. This cluster underscores that transformation is not value-neutral but is directed toward socially meaningful ends. The following six IMMTAs fall under this cluster: Integrated Mixed Methods Child–Parent Research (IMMCPR), Integrated Mixed Methods Participatory Video Research (IMMPVR), Mixed Methods Community-Based Participatory Research (MMCBPR), Mixed Peace Research, Critical Mixed Methods Action Research (CMMAR), and Mixed Methods Impact Evaluation (MMIE) (see Table 1).

For example, Mixed Methods Community-Based Participatory Research (MMCBPR) fully integrates collaborative qualitative inquiry with quantitative measures for co-produced knowledge to promote social change (DeJonckheere et al., 2019). Within this approach, integration explicitly is oriented toward addressing issues of power and inequality by positioning community members as active partners in the research process rather than as passive participants. This collaborative structure enables the inclusion and amplification of voices that often are underserved, marginalized, oppressed, disenfranchised, or silenced, ensuring that their perspectives shape both the generation and interpretation of data. Qualitative insights derived from community engagement are brought into dynamic interaction with quantitative findings, allowing lived experiences to inform the meaning of statistical patterns while quantitative results provide leverage for advocacy and policy change. In this way, dominant narratives can be challenged and reinterpreted through the inclusion of alternative perspectives that might otherwise remain excluded. Consequently, transformation is not limited to methodological integration (Cabrera and Reiner, 2018; Collingridge, 2013; Driscoll et al., 2007; Isaac et al., 2016; Kerrigan, 2014; Leal et al., 2018; Miles and Huberman, 1994; Sandelowski et al., 2009; Tashakkori and Teddlie, 1998; Weaver-Hightower, 2014) but extends to epistemological and ethical reorientation, as knowledge is co-constructed in ways that are accountable to the communities that it represents and directed toward equitable and socially meaningful outcomes.

Similarly, Integrated Mixed Methods Child–Parent Research (IMMCPR) builds on child–parent research, wherein child and parent as co-researchers within a dialogic, participatory, and ethically grounded framework that centers children’s voices as legitimate and authoritative sources of knowledge (Abrams et al., 2020; Abrams and Schaefer, 2022; Schaefer et al., 2020, 2021). With IMMCPR, integration further supports the ethos of the approach, which is oriented toward addressing power asymmetries inherent in adult–child relationships by working to flatten traditional hierarchical structures and to establish conditions of ethical symmetry. Children, parents, and grandparents are engaged not as objects of study, but as active contributors across the research process—from ideation and data collection to analysis and dissemination—thereby ensuring that their perspectives directly inform the construction and interpretation of findings (Schamroth et al., 2026). IMMCPR emphasizes how qualitative and quantitative forms of data are brought into interaction in ways that reflect children’s lived experiences, interests, and meaning-making processes, while also enabling broader patterns to be identified and contextualized. Such an approach was evident when M. L. Abrams tracked not only the amount of time the child–parent research team dedicated to preparing for the AERA presentation, but also the amount of time each of the five children (herself included) responded to questions A. J. Onwuegbuzie asked them in debriefing style (Abrams et al., 2021). Pairing time distribution with interview transcripts and video data to explore aspects of equity is one form of integration, even though the approach was not fully integrated at the time.

Through this collaborative and recursive engagement, dominant adult-centric narratives are challenged, and new forms of knowledge emerge that are dialogically co-constructed and socially responsive. IMMCPR also creates space for intersectional identities to emerge within and among child–parent research teams (cf. Kabuto, 2022), wherein power structures of parenthood, of academia, and of privilege can be examined more critically. Consequently, transformation extends beyond methodological integration (Cabrera and Reiner, 2018; Collingridge, 2013; Driscoll et al., 2007; Isaac et al., 2016; Kerrigan, 2014; Leal et al., 2018; Miles and Huberman, 1994; Sandelowski et al., 2009; Tashakkori and Teddlie, 1998; Weaver-Hightower, 2014) to encompass epistemological and ethical reconfiguration, as research becomes a site for empowering youth voice, for fostering relational accountability, and for advancing more equitable and inclusive forms of inquiry.

#### Emergence and complexity

The fifth cluster focuses on the emergent and nonlinear nature of knowledge production. IMMTAs within this domain recognize that integration does not follow a predetermined path but unfolds through complex interactions among data, methods, and interpretations. Emergence is understood as a defining feature of mixed methods inquiry, wherein new insights arise from the recursive engagement of multiple perspectives. This cluster aligns closely with the concept of meta-inference, emphasizing that knowledge is generated through interaction rather than aggregation. The following seven IMMTAs fall under this cluster: Mixed Methods Visual Displays (MMVDs), Mixed Methods Grounded Theory (MM-GT), Integrated Mixed Methods Systematic Reviews (IMMSR), Mixed Methods Bibliometrics (MMB), Mixed Methods Randomized Controlled Trials (RCTs), Quasi-Experimental Mixed Methods (QEMM), and Transformational Assessment (see Table 1).

For example, Integrated Mixed Methods Systematic Reviews (IMMSR) fully integrate systematic reviews of quantitative research and qualitative research studies by synthesizing evidence across studies for comprehensive evidence synthesis. Within this approach, emergence unfolds through the interaction of diverse forms of evidence, as qualitative findings (e.g., thematic interpretations, contextual insights) and quantitative results (e.g., effect sizes, statistical trends) are iteratively compared, contrasted, and reinterpreted. Rather than aggregating findings, IMMSR facilitates the recursive re-examination of evidence across studies, enabling new patterns and relationships to emerge that are not visible within individual datasets. These emergent insights are expressed through meta-inferences that integrate meaning and magnitude, context and generalizability, thereby producing a more comprehensive and nuanced understanding of the phenomenon under investigation (Onwuegbuzie, 2026).

Relatedly, when addressing IMMSR, researchers might consider the use of bibliometrics (i.e., MMB). Especially given the prolific embeddedness and use of artificial intelligence (AI), researchers embracing integrated mixed methods bibliometrics (IMMB) can inform and build on emerging uses of AI in mixed methods literature reviews. In particular, the Mixed-Methods & AI (MIXAI) approach (Costa et al., 2025) includes chain-of-thought (CoT) prompting with large language models (LLMs) to confirm, to clarify, and/or to challenge the results from manual searches and thematic assessments of data, inclusive of the generation of tables.

When it comes to AI, which is ever-evolving, IMMTAs can offer researchers rigorous and flexible approaches to embrace, to experiment with, and to problematize how full(er) integration occurs with the help of LLMs. The key is transparency. Barfar et al. (2026) uploaded their datasets to the Harvard Dataverse Repository (Barfar et al., 2025), as well as included the code notebook for researchers interested in adopting and implementing the reproducible code (Barfar, 2025). Although such transparency does not guarantee a full(er) integration, it can, however, showcase how and when integration occurs and in what ways the mixed methods research involves data transformation through such integration. Furthermore, given that research of AI in education has excluded psychological implications and applications especially with regard to equity (Ni and Li, 2026), the CDP-guided nature of IMMTAs make them both germane and essential to the field of psychological sciences.

More broadly, these developments illustrate how the meta-principles of CDP 2.0 can guide the responsible integration of emerging technologies into psychological research. From the perspective of *criticality*, researchers should interrogate how AI systems might reproduce existing biases, might amplify inequities, might privilege particular forms of knowledge, or might obscure important contextual nuances. From the perspective of *dialectics*, AI-generated outputs should not be treated as authoritative endpoints but, rather, as additional representations that are recursively compared, challenged, refined, and synthesized alongside human interpretations. Finally, *pluralism* encourages researchers to integrate AI-assisted analyses with diverse methodological, theoretical, and partner perspectives, recognizing that AI constitutes one valuable analytic resource rather than a replacement for human judgment. Consequently, CDP 2.0 provides a philosophical framework for engaging emerging technologies critically, reflexively, transparently, and ethically while preserving the transformative and integrative aims of IMMTA.

Similarly, Mixed Methods Randomized Controlled Trials (RCTs) fully integrate experimental designs with qualitative process data to explain causal mechanisms and to evaluate intervention impacts (Edmunds et al., 2022). In this context, emergence arises through the interaction between quantitative estimates of effect and qualitative accounts of implementation, experience, and context. Qualitative data are used to reinterpret and to contextualize statistical outcomes, while quantitative findings inform the significance and scope of qualitative insights. Through iterative engagement, researchers move beyond simple cause–effect conclusions to generate meta-inferences that explain not only whether an intervention works, but how, for whom, and under what conditions, thereby producing knowledge that is both explanatory and contextually grounded.

Transformational Assessment likewise exemplifies emergence through the recursive integration of qualitative research and quantitative research methodologies critically to examine, to reconstruct, and to refine assessment systems. In this approach, multiple forms of data—such as performance metrics, experiential accounts, and contextual indicators—are brought into sustained interaction, allowing dominant assessment frameworks to be interrogated and reconfigured. Through iterative cycles of analysis, interpretation, and redesign, new evaluative constructs and criteria emerge that more accurately reflect diverse human experiences. These meta-inferences are not derived from aggregation but from the dynamic interplay of perspectives, ensuring that assessment systems evolve in ways that are both analytically rigorous and socially responsive (Onwuegbuzie and Abrams, 2026a).

#### Integrating the five conceptual clusters

Together, these five clusters provide a coherent structure for understanding IMMTA as a meta-transformative methodological framework. They capture the key dimensions of transformation—epistemic, methodological, reflexive, ethical, and emergent—while offering practical guidance for designing and conducting mixed methods research. By organizing the 26 IMMTAs in this way, researchers can more readily identify pathways for integrating diverse approaches and for enacting the principles of CDP 2.0 in their work. Accordingly, the five clusters should be interpreted as overlapping dimensions of methodological emphasis rather than as mutually exclusive categories, with individual IMMTAs often spanning multiple clusters while differing only in the aspects of CDP 2.0 that they foreground operationally.

Importantly, the examples presented throughout these five clusters are intended to illustrate, rather than to review exhaustively, emerging directions in mixed methods research within psychological science. Collectively, these developments—including advances in AI-assisted inquiry, integrated design-based research, participatory and justice-oriented approaches, reflexive methodologies, increasingly recursive forms of integration, and emerging applications addressing equity in psychological research—reflect a broader movement toward more dynamic, interconnected, and ethically responsive research practices. As noted earlier, recent scholarship has highlighted that AI research in education largely has excluded psychological implications and applications, particularly with respect to equity (Ni and Li, 2026). This observation further underscores the importance of CDP 2.0-guided IMMTAs for psychological science because the principles of criticality, dialectics, and pluralism provide a coherent philosophical basis for integrating methodological innovation with ethical responsibility, epistemic inclusivity, analytic rigor, and equitable knowledge production. Accordingly, we contend that CDP 2.0 provides a unifying meta-philosophical framework capable of accommodating, explaining, and extending these emerging methodological developments across diverse domains of psychological research.

The present article intentionally illustrates the operationalization of IMMTA through multiple methodological exemplars distributed across the five conceptual clusters rather than through a single extended empirical case study. This approach reflects the article’s primary objective as a methodological theory paper: to demonstrate the generalizability of the representational–recursive framework across diverse forms of mixed methods research rather than to validate it within one specific application. Accordingly, the examples drawn from published and emerging scholarship are intended to show how recursive integration, transformatizing, and meta-inference can be enacted across multiple methodological contexts. We view future empirical studies applying IMMTAs in specific psychological investigations as an important next phase in the ongoing development, refinement, and evaluation of the framework.

### The representational-recursive paradigm

The representational–recursive paradigm provides the analytic infrastructure through which the meta-transformative aims of IMMTA can be enacted. Whereas IMMTA reconceptualizes integration as a continuous and transformative process, the representational–recursive paradigm specifies the mechanisms through which such transformation unfolds at the level of data, analysis, and inference. Central to this paradigm is a shift from viewing data as fixed entities to understanding them as representations that are constructed, transformed, and reinterpreted through iterative analytic engagement. Integration, in this context, is not achieved by combining discrete datasets but by recursively re-representing them across analytic forms, thereby generating emergent insights.

#### Representational ontology

A foundational premise of the representational–recursive paradigm is that what researchers commonly refer to as *data* are not neutral or self-evident entities but are *constructed representations*. Data are produced through processes of selection, abstraction, coding, measurement, and interpretation—all of which are shaped by conceptual frameworks, theoretical frameworks, methodological decisions, and contextual conditions. Whether expressed as numerical values, textual narratives, visual images, or categorical codes, data are always already mediated by representational choices that influence what can be known and how it can be known (Kitchin, 2014).

From this perspective, the distinction between qualitative and quantitative data is not ontological but representational. Therefore, representational ontology should be understood as an epistemological stance concerning the analytic status of research representations rather than as an ontological claim about the independent nature of reality itself. Qualitative data do not inherently possess meaning independent of interpretation, nor are quantitative data intrinsically objective or free from context. Rather, both forms of data are constructed through different representational logics: qualitative approaches often emphasize contextual richness, narrative structure, and interpretive depth, whereas quantitative approaches prioritize abstraction, standardization, and comparability. These differences are not markers of incompatibility but of *alternative representational strategies* for engaging with phenomena.

By reframing data as representations, the representational–recursive paradigm dissolves the traditional qualitative–quantitative divide. Integration is no longer a matter of reconciling fundamentally different types of data but of translating and transforming representations across analytic forms. This shift enables a more flexible and dynamic approach to mixed methods research, in which qualitative and quantitative elements can be brought into relation through processes of re-representation rather than through juxtaposition alone.

Importantly, this ontological stance aligns with broader developments in the philosophy of science and data studies, which emphasize the constructed and situated nature of knowledge (Leonelli, 2019). It also provides the conceptual foundation for the processes of transformation and recursion that characterize the representational–recursive paradigm.

#### Transformatizing

Building on a representational ontology, the paradigm centers on *transformatizing* as the primary analytic process through which integration is achieved. Transformatizing refers to the *multidirectional transformation of data across representational forms* (Onwuegbuzie, 2026), encompassing both *quantitizing* (the transformation of qualitative data into quantitative representations) and *qualitizing* (the transformation of quantitative data into qualitative representations). Unlike traditional approaches of quantitzing (Collingridge, 2013; Isaac et al., 2016; Kerrigan, 2014; Leal et al., 2018; Miles and Huberman, 1994; Sandelowski et al., 2009; Tashakkori and Teddlie, 1998; Weaver-Hightower, 2014) and qualitizing (Tashakkori and Teddlie, 1998) that treat these processes as auxiliary techniques, transformatizing positions them as core mechanisms of integration.

Crucially, transformatizing is not a unidirectional or terminal process. Rather, it is *iterative and multidirectional*, allowing data to move back and forth across representational forms in response to emerging analytic insights. For example, qualitative themes may be quantified to examine patterns or relationships, which, in turn, may be reinterpreted qualitatively to explore contextual nuances. This movement is not merely technical but interpretive, involving shifts in perspective that enable new ways of understanding the phenomenon under study.

Through transformatizing, qualitative and quantitative representations are placed in dynamic relation, allowing each to inform and to reshape the other. This process aligns with calls for more integrative approaches to mixed methods analysis that move beyond parallel or sequential designs toward deeper forms of interaction (Schoonenboom, 2022, 2023). However, transformatizing extends these approaches by emphasizing the recursive and transformative nature of integration, rather than treating it as a discrete analytic step.

Moreover, transformatizing enables the exploration of tensions and inconsistencies among representations. Rather than seeking convergence as the primary goal, it allows for the identification and examination of divergence, contradiction, and complexity. These moments of tension are not treated as problems to be resolved, but as opportunities for deeper insight, consistent with the dialectical orientation of CDP 2.0.

#### Recursive analytic cycles

The transformative potential of transformatizing is realized through *recursive analytic cycles*, which structure the iterative movement across representations. Recursion refers to the process by which outputs of one analytic stage become inputs for subsequent stages, creating a feedback loop that enables continuous refinement and reinterpretation. In the context of mixed methods research, recursive cycles involve repeated engagement with data across qualitative and quantitative forms, allowing insights to evolve over time.

These cycles are characterized by three interrelated features: *iteration*, *reflexivity*, and *deep integration*. Iteration involves revisiting data and analytic decisions multiple times, each time incorporating new insights or perspectives. This iterative process contrasts with linear models of analysis, in which data are processed in a fixed sequence leading to a final result. Instead, recursion allows for the ongoing development of understanding, with each cycle contributing to a more nuanced and comprehensive interpretation.

Reflexivity plays a central role in recursive cycles by foregrounding the researcher’s involvement in the analytic process. As representations are transformed and reinterpreted, researchers must continually examine how their assumptions, decisions, and positionalities influence the analysis. This reflexive engagement ensures that recursion is not merely mechanical, but critically informed, aligning with the principles of CDP 2.0.

Deep integration emerges from the sustained interaction of representations across cycles. Rather than achieving integration at a single point, recursive analysis enables integration to develop progressively, as insights from one representation inform and reshape others. This process results in a form of integration that is not only more comprehensive, but also more responsive to complexity and context.

Recursive analytic cycles thus provide the structural mechanism through which transformatizing operates, enabling the continuous transformation and integration of representations. They also align with broader methodological movements that emphasize iteration and reflexivity as key components of rigorous qualitative research and mixed methods research (Srivastava and Hopwood, 2009).

#### Meta-inference

The culmination of representational–recursive analysis is *meta-inference*, representing the generation of knowledge that emerges from the interaction of multiple representations. Unlike traditional notions of inference, which often involve drawing conclusions from a single dataset or analytic approach, meta-inference inherently is integrative and emergent. It arises not from the aggregation of findings, but from their *interaction within recursive analytic processes* (Schoonenboom, 2022).

Meta-inference is characterized by its capacity to produce insights that are qualitatively different from those derived from individual representations (Tashakkori and Teddlie, 1998). Through the iterative transformation and comparison of representations, new patterns, relationships, and interpretations become visible. These insights are not reducible to any single representation but are co-constructed through their interaction (Polyviou et al., 2024; Schoonenboom, 2022, 2023; Shannon-Baker and Hunt-Anderson, 2025; Younas and Durante, 2022, 2023; Younas et al., 2023, 2025). In this sense, meta-inference embodies the principle of emergence, whereby complex outcomes arise from the dynamic interplay of simpler elements.

Importantly, meta-inference does not require convergence among representations. Divergence, contradiction, and complexity are integral to the process, providing opportunities for deeper understanding. For example, discrepancies between qualitative narratives and quantitative patterns may reveal underlying contextual factors or highlight limitations in measurement. By engaging these differences rather than resolving them prematurely, meta-inference supports a more nuanced and contextually grounded form of knowledge production. This interaction-based conception of integration aligns with and extends existing frameworks in mixed methods research, which emphasize the importance of integrating findings at multiple stages (Polyviou et al., 2024; Schoonenboom, 2022, 2023; Shannon-Baker and Hunt-Anderson, 2025; Younas and Durante, 2022, 2023; Younas et al., 2023, 2025). However, the representational–recursive paradigm advances this perspective by specifying the mechanisms through which integration leads to emergent knowledge. Meta-inference is thus not a final step but the outcome of an ongoing process of transformation and recursion.

#### Operationalizing IMMTA through representational–recursive analysis

Taken together, representational ontology, transformatizing, recursive analytic cycles, and meta-inference constitute a coherent analytic framework for operationalizing IMMTA. By conceptualizing data as representations, the paradigm dissolves traditional methodological divides and enables flexible movement across analytic forms. Through transformatizing, representations are transformed multidirectionally, allowing for dynamic interaction. Recursive cycles structure this interaction, supporting iterative refinement and deep integration. Finally, meta-inference captures the emergent knowledge that arises from these processes, providing a basis for understanding integration as a system-level property of inquiry.

In this way, the representational–recursive paradigm provides the analytic infrastructure necessary to enact the meta-transformative vision of IMMTA. It translates philosophical commitments into methodological practices, enabling integration to function not as an isolated task but as an ongoing, generative process. As with other newly proposed methodological frameworks, the utility, scope, and boundary conditions of the present framework ultimately will require continued empirical application, methodological refinement, and critical evaluation across diverse psychological research contexts. This shift establishes the conditions for a unified paradigm in which philosophical grounding, methodological design, and analytic practice are aligned coherently.

### Integrating CDP 2.0, IMMTA, and the representational-recursive framework

The preceding sections have articulated three interrelated components: Critical Dialectical Pluralism (CDP 2.0) as a meta-transformative and multidimensional metaparadigmatic and meta-philosophical foundation, the Integrated Mixed Methods Transformation Approach (IMMTA) as a meta-transformative methodological framework, and the representational–recursive paradigm as an analytic infrastructure. This section synthesizes these components into a unified methodological system by mapping their conceptual correspondences and demonstrating how they operate across philosophical, methodological, and analytic levels of inquiry. The central claim advanced here is that *CDP 2.0, IMMTA, and the representational–recursive paradigm are not discrete frameworks but interdependent layers of a single, unified paradigm of mixed methods research*.

At a structural level, CDP 2.0 provides the *philosophical grounding*, IMMTA offers the *methodological articulation*, and the representational–recursive paradigm specifies the *analytic mechanisms* through which transformation and integration are enacted. As summarized in Table 1, IMMTAs operationalize CDP principles at the methodological level, which are then enacted analytically through representational–recursive processes. This layered relationship enables a coherent alignment among epistemology, methodology, and method, addressing a persistent limitation in mixed methods research: the lack of integration across these levels. As shown in Table 1, each IMMTA can be understood as operationalizing one or more CDP principles, thereby linking philosophical commitments to methodological enactment. By linking these dimensions, the unified paradigm advances a conception of mixed methods inquiry in which philosophical commitments directly are operationalized through methodological design and analytic practice.

#### Mapping CDP 2.0 to IMMTA and representational mechanisms

The integration of these frameworks can be understood through the alignment of CDP 2.0’s three meta-principles—criticality, dialectics, and pluralism—with corresponding IMMTA features and representational–recursive mechanisms. This mapping provides a systematic account of how philosophical principles are translated into methodological and analytic processes.

#### Criticality → justice-oriented inquiry → inclusive representation

The principle of criticality foregrounds issues of power, marginalization, and representation in knowledge production. Within IMMTA, this principle is operationalized through *justice-oriented inquiry*, which emphasizes the ethical dimensions of research, including the inclusion of diverse voices and the pursuit of equitable outcomes. At the analytic level, this orientation is enacted through *inclusive representation*, whereby data are constructed, transformed, and interpreted in ways that reflect multiple perspectives and resist epistemic exclusion.

Inclusive representation extends beyond sampling or participation to encompass the entire analytic process. It involves deliberate efforts to ensure that representations do not privilege dominant narratives or obscure underserved, marginalized, oppressed, disenfranchised, and silenced experiences. For example, qualitative narratives may be transformed into quantitative indicators in ways that preserve contextual nuance, while quantitative patterns may be reinterpreted qualitatively to surface underlying inequities. In this sense, inclusion is not merely a design feature but an analytic practice, embedded within processes of representation and transformation.

This alignment illustrates how criticality moves from a philosophical commitment to an actionable methodological and analytic principle. By embedding justice-oriented considerations within representational processes, the unified paradigm ensures that integration is not only technically rigorous, but also ethically grounded. In so doing, it reinforces the inseparability of epistemic rigor and ethical responsibility within mixed methods inquiry.

#### Dialectics → transformative integration → recursive transformation

Dialectics, as articulated within CDP 2.0, emphasizes the productive engagement of difference through interaction and synthesis. Within IMMTA, this principle is expressed as *transformative integration*, wherein qualitative and quantitative elements are not merely combined but dynamically transformed through their interaction. At the analytic level, this process is operationalized through *recursive transformation*, which involves iterative cycles of re-representation across analytic forms.

Recursive transformation captures the dynamic interplay among representations, allowing them to inform, to challenge, and to reshape one another. For instance, a quantitative trend may prompt a re-examination of qualitative data, leading to the identification of new themes, which, in turn, may be quantified to explore their distribution. This iterative movement reflects a dialectical process in which contradictions and divergences are not resolved prematurely but are explored as sources of insight.

Importantly, recursive transformation redefines integration as an ongoing process rather than a discrete stage. It aligns with the dialectical view that synthesis emerges through interaction, not through the imposition of coherence. By structuring analysis as a series of recursive cycles, the paradigm enables the continuous transformation of representations, resulting in deeper and more nuanced understandings.

This alignment demonstrates how dialectics is translated into methodological and analytic practice, providing a mechanism for engaging complexity and difference in mixed methods research. In so doing, it positions recursive transformation as the analytic engine through which integration becomes *generative* rather than merely combinatory.

#### Pluralism → epistemic plurality → representational ontology

Pluralism, the third meta-principle of CDP 2.0, affirms the legitimacy of multiple ways of knowing and rejects hierarchical epistemologies. Within IMMTA, this principle is operationalized as *epistemic plurality*, which encourages the inclusion and integration of diverse methodological and analytic perspectives. At the analytic level, pluralism is grounded in a *representational ontology*, which conceptualizes data as constructed representations rather than as inherently distinct qualitative or quantitative entities.

Representational ontology provides the conceptual basis for engaging multiple forms of data without privileging one over another. By framing qualitative and quantitative data as alternative representations of phenomena, it enables their integration through processes of transformation and translation. This perspective dissolves traditional methodological divides and supports the coexistence of multiple analytic approaches within a single study.

Epistemic plurality, in this sense, is not simply about using multiple methods but about engaging multiple forms of representation in a coherent and integrative manner. It allows researchers to draw on the strengths of different approaches while maintaining a commitment to inclusivity and coherence. Through representational ontology, pluralism becomes operational, providing a foundation for the dynamic interaction of representations that characterizes the representational–recursive paradigm.

#### Toward a unified paradigm of mixed methods research

The alignment of CDP 2.0, IMMTA, and the representational–recursive paradigm yields a unified framework in which philosophical principles, methodological orientations, and analytic practices are mutually reinforcing. This integration addresses a central challenge in mixed methods research: the tendency for philosophical, methodological, and analytic dimensions to remain disconnected or only loosely aligned (Bryman, 2006; Collins, 2018; Creamer, 2018; Fetters et al., 2013; Fetters and Freshwater, 2015; Poth, 2018, 2022). Within the unified paradigm, integration is understood as a *system-level property of inquiry*, emerging from the interaction of representations within a recursive analytic process. This conception contrasts with traditional models that treat integration as a discrete task, such as merging datasets or comparing findings. Instead, integration is continuous, iterative, and transformative, shaping and being shaped by all aspects of the research process.

This paradigm also redefines the role of the researcher. Rather than acting as a neutral observer who combines independent strands of data, the researcher becomes an active participant in the construction and transformation of representations. Therefore, reflexivity is integral to the process, ensuring that analytic decisions are examined critically and that multiple perspectives are engaged.

Moreover, the unified paradigm expands the scope of mixed methods research by incorporating ethical and epistemic considerations into its core. Through the integration of CDP 2.0, issues of power, inclusion, and justice are embedded within methodological and analytic practices, rather than treated as external concerns. This alignment enhances the capacity of mixed methods research to address complex social phenomena—including wicked problems (Mertens et al., 2016) and super wicked problems (Levin et al., 2012)—in ways that are both rigorous and socially responsive. These capabilities are especially important in psychological science, wherein researchers frequently seek to understand phenomena that emerge across interacting individual, relational, cultural, institutional, and societal levels of analysis.

#### Integration as emergent, interaction-based knowledge production

A key implication of this unified paradigm is the reconceptualization of integration as an *emergent, interaction-based process of knowledge production*. Rather than viewing integration as the combination or synthesis of pre-existing findings, it is understood as the outcome of dynamic interactions among representations within recursive analytic cycles. This perspective aligns with theories of emergence, which emphasize how complex outcomes arise from the interaction of simpler elements rather than from their aggregation (Baggio and Parravicini, 2019; Goldstein, 1999; Sawyer, 2005).

In this framework, knowledge is not merely discovered or assembled, but co-constructed through iterative engagement with data. Meta-inference, as discussed in the previous section, becomes the primary mechanism through which this knowledge is generated. It reflects the culmination of recursive transformations, capturing insights that are not reducible to any single representation.

Importantly, this conception of integration accommodates complexity, divergence, and uncertainty. Rather than seeking convergence as the primary indicator of rigor, it recognizes that differences among representations can provide valuable insights. By engaging these differences dialectically, the unified paradigm supports a more nuanced and contextually grounded form of inquiry.

#### Implications for methodological theory

The integration of CDP 2.0, IMMTA, and the representational–recursive paradigm has several implications for the development of mixed methods methodology. First, it advances a *multi-level framework* that links philosophical, methodological, and analytic dimensions, providing a more coherent foundation for mixed methods research. Second, it shifts the focus from methodological combination to *transformative integration*, emphasizing the dynamic and emergent nature of inquiry. Third, it embeds ethical considerations within analytic processes, ensuring that issues of power and inclusion are integral to research design and implementation.

By articulating these connections, the unified paradigm contributes to ongoing efforts to refine and to extend mixed methods research as a distinct and evolving field. It provides a conceptual and practical foundation for conducting research that is not only methodologically rigorous, but also epistemically inclusive and socially responsive. In so doing, it advances a re-envisioning of mixed methods inquiry as an integrative, reflexive, and transformative enterprise.

Although the unified paradigm offers a comprehensive framework, its implementation may present challenges, including the analytic complexity associated with recursive transformations, the need for methodological expertise across multiple approaches, and the demands of maintaining coherence across iterative cycles. Accordingly, future research should focus on developing strategies for operationalizing these processes in applied contexts.

#### Establishing a unified paradigm for transformative mixed methods inquiry

In sum, the integration of CDP 2.0, IMMTA, and the representational–recursive paradigm establishes a unified approach to mixed methods research in which philosophical commitments, methodological strategies, and analytic processes are fully aligned. Through the mapping of criticality, dialectics, and pluralism onto justice-oriented inquiry, transformative integration, and epistemic plurality—and their corresponding representational mechanisms—this framework demonstrates how integration can be enacted as a continuous, recursive, and transformative process. As such, it advances a new paradigm in which mixed methods research is defined not by the combination of approaches but by the dynamic interaction of representations that generate emergent, transformative knowledge.

### Methodological implications

The integration of CDP 2.0, IMMTA, and the representational–recursive paradigm has substantive implications for how mixed methods research is designed, conducted, and evaluated. By reconceptualizing integration as a continuous, recursive, and transformative process grounded in ethical and epistemic commitments, this unified framework reshapes each stage of the research process. The following subsections outline these implications across research design, data collection, data analysis, validity and legitimation, and ethical practice.

#### Key recommendations for researchers

In order to facilitate application of the unified framework, we offer the following practical recommendations:

- Design for integration from the outset. Treat integration as a guiding design principle rather than as a step that occurs after qualitative research and quantitative research components/phases/strands have been completed.
- Work recursively across representations. Allow qualitative representations, quantitative representations, visual displays, and emerging interpretations to inform one another continuously through iterative cycles of transformatizing.
- Build reflexivity into every stage of inquiry. Revisit research questions, analytic decisions, positionality, and ethical commitments throughout the research process rather than viewing them as fixed at the design stage.
- Generate and evaluate meta-inferences iteratively. Develop overarching interpretations progressively by integrating evolving representations instead of waiting until the conclusion of the study to synthesize findings.
- Align methodological decisions with philosophical commitments. Select and operationalize IMMTAs in ways that explicitly reflect the CDP 2.0 principles of criticality, dialectics, and pluralism while maintaining transparency regarding how transformation occurs throughout the inquiry.

#### Research design

Within the unified paradigm, research design is no longer conceived as the selection and sequencing of distinct qualitative and quantitative components but as the *configuration of a dynamic system of inquiry*. This system is structured around *transformation* rather than addition, requiring that integration be embedded from the outset rather than introduced at later stages. Design decisions can be guided by the CDP 2.0 principles embodied in specific IMMTAs (Table 1), allowing researchers to align philosophical commitments with methodological choices.

A key implication is that research questions must be formulated in ways that *invite multiple forms of representation and iterative engagement*. Rather than aligning questions with specific methods (e.g., qualitative for exploration, quantitative for explanation), questions are designed to accommodate transformation across representations. This orientation encourages flexibility in design, allowing researchers to adapt their strategies in response to emerging insights.

Designs within this framework inherently are *iterative and adaptive*, often involving cycles of data generation and analysis that inform subsequent stages. This contrasts with linear models in which design decisions are fixed in advance. Instead, researchers anticipate transformation, building in opportunities for revisiting and refining research questions, data sources, and analytic strategies.

Moreover, integration is conceptualized as a *design principle rather than as a procedural step*. This aligns with integrative design approaches that emphasize planning for interaction across methods (Fetters et al., 2013), but extends them by foregrounding transformation and recursion as central features. As a result, research design becomes a site of epistemic and methodological innovation, wherein the conditions for emergent knowledge are deliberately constructed.

#### Data collection

The reconceptualization of data as representations has significant implications for data collection. Rather than treating qualitative and quantitative data as distinct entities to be collected separately, data collection is understood as the *generation of multiple, interrelated representations of phenomena*.

This perspective encourages the use of *complementary and overlapping data sources*, with attention to how different forms of representation can inform one another. For example, survey data may be designed not only to capture measurable variables, but also to facilitate subsequent qualitative interpretation, while qualitative interviews may be structured in ways that support transformation into quantitative forms.

Importantly, data collection becomes *responsive and iterative*, informed by ongoing analytic processes. Early analyses may reveal gaps, tensions, or unexpected patterns that prompt the collection of additional data or the refinement of existing instruments. This iterative approach aligns with recursive analytic cycles, ensuring that data generation and analysis are closely integrated.

The unified paradigm also emphasizes the importance of *inclusive data practices*, consistent with the critical and justice-oriented commitments of CDP 2.0. This includes engaging participants as contributors to knowledge production, incorporating diverse perspectives, and attending to the contexts in which data are generated. Thus, data collection is not merely a technical process, but a relational one, shaped by interactions among researchers, participants, and contexts.

#### Data analysis

Data analysis within the unified paradigm is characterized by *transformatizing and recursive engagement with representations*. Rather than analyzing qualitative and quantitative data separately and then integrating results, analysis involves continuous movement across representational forms. This process enables the generation of meta-inferences through the dynamic interaction of representations, yielding insights that would not emerge from isolated analytic approaches.

This process is structured through *recursive analytic cycles*, in which representations are iteratively transformed, compared, and reinterpreted. For example, qualitative themes may be quantified to examine patterns, which may then be qualitatively reinterpreted to explore context and meaning. Each cycle generates new insights that inform subsequent transformations, leading to progressively deeper integration.

A central implication is that analysis becomes *processual rather than stage-based*. Integration is not confined to a specific phase but occurs throughout the analytic process, shaping how data are interpreted at each step. This aligns with integrative approaches to mixed methods analysis but extends them by specifying the mechanisms—transformatizing and recursion—through which integration is achieved.

Moreover, analysis is inherently *reflexive*, requiring ongoing examination of how analytic decisions influence representations and interpretations. Reflexivity is not treated as an adjunct to analysis but as an integral component, ensuring that transformations are critically informed and contextually grounded (Srivastava and Hopwood, 2009).

Through these processes, data analysis becomes a site of *emergent knowledge production*, wherein insights arise from the interaction of representations rather than from their aggregation.

#### Validity and legitimation

The unified paradigm necessitates a rethinking of validity and legitimation in mixed methods research. Traditional criteria, often derived from either qualitative or quantitative paradigms, may not fully capture the rigor of integrative and transformative approaches. Instead, validity/legitimation is reconceptualized as *process-oriented and interaction-based*, reflecting the dynamic nature of integration.

One implication is the importance of *integration legitimation*, which assesses the extent to which qualitative and quantitative components are meaningfully related and mutually informative (Onwuegbuzie and Johnson, 2006). Within the representational–recursive paradigm, this extends to evaluating the quality of transformations across representations and the coherence of recursive analytic cycles. This expanded view positions legitimation as an ongoing evaluative process embedded within analysis rather than as a post-hoc assessment.

Within psychological research, these considerations have particular relevance for measurement and construct development. Rather than treating constructs as fixed entities captured fully by a single instrument, the representational–recursive paradigm conceptualizes construct operationalization as an iterative process in which qualitative representations, quantitative indicators, theoretical understandings, and contextual interpretations recursively inform one another. Consequently, evidence for measurement validity is strengthened through the ongoing refinement of constructs and instruments as emerging qualitative insights and quantitative findings are integrated across successive analytic cycles. Likewise, quantitative indices—including effect sizes—should not be interpreted as isolated indicators of practical importance but, rather, as representations whose meaning is enriched through recursive engagement with qualitative evidence, contextual conditions, participant perspectives, and reflexive interpretation. Such an approach enables psychological researchers to move beyond decontextualized statistical interpretation toward more comprehensive, transparent, and theoretically grounded explanations of complex psychological phenomena.

Another implication is the recognition of *emergent legitimation*, whereby the credibility of findings is tied to the transparency and rigor of the processes through which they are generated. This includes documenting how representations are constructed, transformed, and interpreted, as well as how decisions are made throughout the research process. In this sense, legitimation is not a fixed property of findings but an emergent quality of the analytic process itself.

The unified paradigm also supports a *pluralistic approach to legitimation*, incorporating multiple criteria that reflect different dimensions of rigor. For example, quantitative standards of reliability and generalizability may be complemented by qualitative criteria such as credibility and contextual sensitivity. Rather than privileging one set of criteria, the paradigm encourages their integration, consistent with its pluralist orientation.

Ultimately, validity is understood as a function of *coherence, transparency, and reflexivity* within a system of recursive transformations. This perspective aligns with broader efforts to develop integrative frameworks for assessing rigor in mixed methods research (Onwuegbuzie, 2026).

#### Ethical and justice-oriented practice

Ethical considerations are not external to the unified paradigm but are embedded within its philosophical and methodological foundations. The integration of CDP 2.0 ensures that research practices are oriented toward *justice, inclusion, and social responsibility*, with implications for all stages of the research process. In this way, ethical commitment becomes an integral feature of inquiry rather than an external criterion applied after the fact.

In research design and data collection, this involves *inclusive participation and representation*, ensuring that diverse perspectives are not only included, but also meaningfully engaged. In data analysis, it requires attention to how representations may amplify or obscure certain voices, as well as reflexive consideration of the researcher’s role in shaping interpretations. Together, these practices ensure that ethical commitments are enacted throughout the research process rather than confined to discrete stages.

Ethical practice also extends to the interpretation and dissemination of findings, wherein researchers must consider how their work may impact participants and communities. This includes avoiding reductive or decontextualized representations and ensuring that findings are communicated in ways that are accessible and responsive to stakeholders.

Importantly, the unified paradigm frames ethics as an *ongoing, recursive process*, rather than as a set of guidelines applied at discrete points. Ethical considerations are revisited throughout the research process, informed by emerging insights and changing contexts. This approach aligns with the critical dimension of CDP 2.0, emphasizing the responsibility of researchers to engage with issues of power and representation in a sustained and reflexive manner.

#### Toward transformative, recursive, and ethically grounded methodological practice

The methodological implications of integrating CDP 2.0, IMMTA, and the representational–recursive paradigm extend across all stages of the mixed methods research process. By embedding transformation, recursion, and ethical commitment within research design, data collection, analysis, and evaluation, the unified framework provides a comprehensive approach to inquiry. It shifts the focus from methodological combination to *dynamic, interaction-based knowledge production*, enabling researchers to engage complexity in ways that are both analytically rigorous and socially responsive.

## Conclusion

This article has advanced a unified methodological paradigm for mixed methods research by integrating CDP 2.0, IMMTA, and the representational–recursive paradigm. Taken together, these frameworks reposition mixed methods inquiry as a dynamic, integrative, and ethically grounded process in which knowledge is generated through the *iterative interaction of representations* rather than through the aggregation of methodological components.

The central contribution of this work lies in its articulation of a *multi-level alignment across philosophy, methodology, and analytic practice*. The explicit alignment of IMMTAs with CDP 2.0 principles further clarifies how philosophical commitments are translated into methodological and analytic practice. CDP 2.0 provides the meta-philosophical grounding through its principles of criticality, dialectics, and pluralism, ensuring that inquiry is attentive to power, oriented toward transformation, and inclusive of diverse epistemologies. IMMTA translates these principles into a methodological framework that reconceptualizes integration as continuous, recursive, and transformative across all stages of research. In turn, the representational–recursive paradigm specifies the analytic mechanisms—representational ontology, transformatizing, recursive cycles, and meta-inference—through which this transformation is enacted.

By linking these levels, the unified paradigm addresses a persistent limitation in mixed methods research: the fragmentation among philosophical commitments, methodological designs, and analytic procedures. Rather than treating these dimensions as loosely connected, the present framework demonstrates how they can be systematically aligned, yielding a more coherent and robust approach to inquiry. In this context, integration becomes a *system-level property* that emerges from the interaction of representations within recursive analytic processes.

A second contribution is the reconceptualization of integration itself. Moving beyond additive and even synergistic models, the paradigm advances a view of integration as *transformative and emergent*, grounded in dialectical interaction and recursive transformation. This shift has implications for how mixed methods research is designed, conducted, and evaluated, emphasizing flexibility, reflexivity, and responsiveness to complexity. It also expands the scope of mixed methods inquiry by embedding ethical considerations—particularly those related to social justice, inclusion, diversity, equity, and social responsibility—*within* analytic processes, rather than treating them as external concerns.

The framework also contributes to methodological theory by specifying mechanisms through which integrative knowledge is produced. Through processes of transformatizing and recursion, qualitative and quantitative representations are brought into dynamic relation, enabling the generation of meta-inferences that are not reducible to any single analytic form. This interaction-based conception of knowledge production aligns with and extends prior work emphasizing the importance of integration in mixed methods research (Bryman, 2006; Creamer, 2018), while providing a more explicit account of how such integration can be operationalized.

Looking forward, several avenues for further development emerge. One direction involves the continued refinement of analytic techniques that support representational transformation and recursion, including tools for managing and visualizing complex interactions among representations. Another involves empirical applications of the unified paradigm across diverse fields, which can provide insights into its practical utility and adaptability in different research contexts.

A particularly promising area for future work concerns the role of *artificial intelligence (AI) in mixed methods research*. AI-based tools have the potential to augment representational–recursive processes by facilitating large-scale transformations, identifying patterns across representations, and supporting iterative analytic cycles. At the same time, their integration raises important questions regarding transparency, interpretability, and ethical responsibility—issues that align closely with the principles of CDP 2.0. As such, the extension of the unified paradigm to include human–AI co-analysis represents a natural and necessary next step, albeit one that requires careful theoretical and methodological development.

In conclusion, the integration of CDP 2.0, IMMTA, and the representational–recursive paradigm advances mixed methods research toward a more coherent, transformative, and ethically grounded form of inquiry. By reconceptualizing integration as an emergent, recursive, and interaction-based process, this framework provides a foundation for addressing complex phenomena in ways that are both methodologically rigorous and socially responsive. In particular, the framework offers methodological guidance for investigating complex psychological phenomena whose causes, manifestations, and consequences span multiple levels of human experience and social context. As mixed methods research continues to evolve, such integrative paradigms will be essential for realizing its full potential.

## Data Availability

The original contributions presented in the study are included in the article/supplementary material, further inquiries can be directed to the corresponding author.
